# Direct coupling analysis of epistasis in allosteric materials

**DOI:** 10.1371/journal.pcbi.1007630

**Published:** 2020-03-02

**Authors:** Barbara Bravi, Riccardo Ravasio, Carolina Brito, Matthieu Wyart

**Affiliations:** 1 Institute of Physics, École Polytechnique Fédérale de Lausanne, Lausanne, Switzerland; 2 Instituto de Fìsica, Universidade Federal do Rio Grande do Sul, Porto Alegre, Brazil; Rutgers University, UNITED STATES

## Abstract

In allosteric proteins, the binding of a ligand modifies function at a distant active site. Such allosteric pathways can be used as target for drug design, generating considerable interest in inferring them from sequence alignment data. Currently, different methods lead to conflicting results, in particular on the existence of long-range evolutionary couplings between distant amino-acids mediating allostery. Here we propose a resolution of this conundrum, by studying epistasis and its inference in models where an allosteric material is evolved *in silico* to perform a mechanical task. We find in our model the four types of epistasis (Synergistic, Sign, Antagonistic, Saturation), which can be both short or long-range and have a simple mechanical interpretation. We perform a Direct Coupling Analysis (DCA) and find that DCA predicts well the cost of point mutations but is a rather poor generative model. Strikingly, it can predict short-range epistasis but fails to capture long-range epistasis, in consistence with empirical findings. We propose that such failure is generic when function requires subparts to work in concert. We illustrate this idea with a simple model, which suggests that other methods may be better suited to capture long-range effects.

## Author summary

Allostery in proteins is the property of highly specific responses to ligand binding at a distant site. To inform protocols of *de novo* drug design, it is fundamental to understand the impact of mutations on allosteric regulation and whether it can be predicted from evolutionary correlations. In this work we consider allosteric architectures artificially evolved to optimize the cooperativity of binding at allosteric and active site. We first characterize the emergent pattern of epistasis as well as the underlying mechanical phenomena, finding the four types of epistasis (Synergistic, Sign, Antagonistic, Saturation), which can be both short or long-range. The numerical evolution of these allosteric architectures allows us to benchmark Direct Coupling Analysis, a method which relies on co-evolution in sequence data to infer direct evolutionary couplings, in connection to allostery. We show that Direct Coupling Analysis predicts quantitatively point mutation costs but underestimates strong long-range epistasis. We provide an argument, based on a simplified model, illustrating the reasons for this discrepancy. Our analysis suggests neural networks as more promising tool to measure epistasis.

## Introduction

Allosteric regulation in proteins allows for the control of functional activity by ligand binding at a distal allosteric site [1] and its detection could guide drug design [2, 3]. Yet, understanding the principles responsible for allostery remains a challenge. How random mutations dysregulate allosteric communication is a valuable information studied experimentally [4] and computationally [5]. Several analyses have highlighted the non-additivity of mutational effects or *epistasis*. This “interaction” between mutations can span long-range positional combinations [6], results in either beneficial or detrimental effects to fitness [7], and shapes protein evolutionary paths [8]. Given the combinatorial complexity of its characterization, empirical patterns of epistasis are still rather elusive [9–12]. Concomitantly, progress in sequencing has led to an unprecedented increase of availability of data arranged into Multiple Sequence Alignments (MSAs) [13] containing many realizations of the same protein in related species. Different methods have been developed to extract information from sequence variability, e.g. Statistical Coupling Analysis [14, 15] was applied to allostery detection in proteins. It was argued that the allosteric pathway was encoded in spatially extended and connected *sectors*, groups of strongly co-evolving amino-acids, supporting that long-range information on the allosteric pathway is contained in the MSA. Another approach, Direct Couplings Analysis (DCA) [16], aims at inferring evolutionary couplihngs between amino-acids. Direct couplings predict successfully residue contacts [16] so to inform the discovery of new folds [17], allow one to describe evolutionary fitness landscapes [18–22] and correlate with epistasis [23, 24]. In the context of allostery, there is no statistical evidence for the existence of long-range direct couplings that would reveal allosteric channels [25], in apparent contradiction with the existence of extended sectors reported in [15] and the observation of long-range epistasis [6]. It is therefore an open question why a pairwise model should be successful at predicting protein structure, but not long-range functional dependencies. In this work we propose an explanation for this discrepancy, by benchmarking DCA in models of protein allostery where a material evolves *in silico* to achieve an “allosteric” task [26–32]. We consider recent models incorporating elasticity [27–30, 32], in which long-range co-evolution [29], elongated sectors [29] and long-range epistasis [32] are present and can be interpreted in terms of the propagation of an elastic signal [32]. We focus on materials evolved to optimize cooperative binding over large distances [30], and find that the four types of epistasis (Synergistic, Sign, Antagonistic, Saturation) exist over a wide spatial range. We perform DCA and find that it predicts well the cost of point mutations but is a rather poor generative model. It can predict short-range epistasis but fails to capture long-range effects, in agreement with empirical findings [25]. Moreover, we test this result for one allosteric protein, the PDZ domain, where epistasis was experimentally measured in [12] along with the inference of DCA energetic couplings, showing support for our prediction. We illustrate why it may be so via a simple model, which suggests that neural networks may be better suited than DCA to capture long-range effects.

### Model for the evolution of allostery

**Fig 1:**
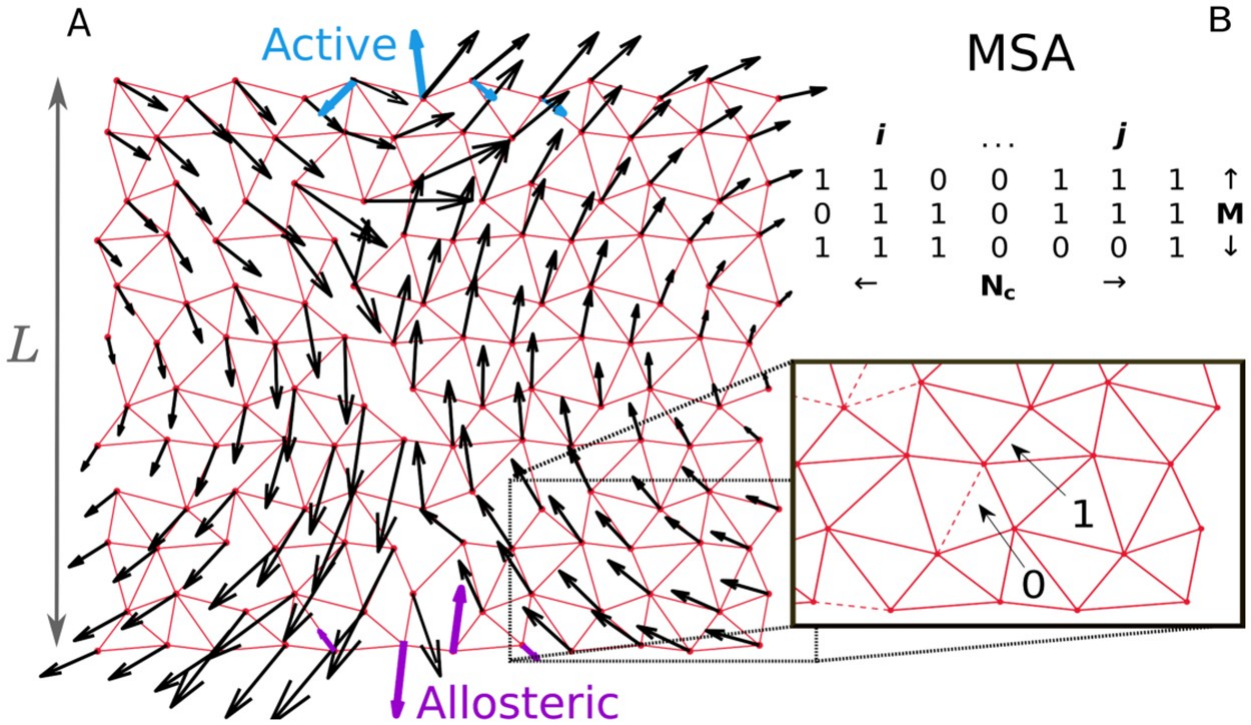
Study of co-evolution in artificial allosteric networks. A: Example of an elastic network made of harmonic springs (red) evolved *in silico* to maximize the cooperativity between the allosteric site (purple) and the active site (blue). The response to binding at the allosteric site is indicated by black arrows, and is found to follow a shear motion. B: Each network corresponds to a sequence of 0 and 1 coding for the spring absence or presence. Our scheme allows us to generate a large number *M* of such sequences, each corresponding to a slightly different shear architecture.

We follow the scheme of [29, 30] where a protein is described by an elastic network of size *L* made of harmonic springs of unit stiffness (here we consider *L* = 12). Binding events are modeled as imposed displacements either at the “allosteric” or at the “active” site (each consisting of several nodes), as shown in color in Fig 1A. Such imposed displacements elicit an elastic response in the entire protein and cost some elastic energy, which defines our binding energy (see Sec. 1 in S1 Text). Following [30], the fitness F measures the cooperativity of binding between allosteric and active site and is defined as the energy difference $F\equiv E^{Ac}-\left(E^{Ac,Al}-E^{Al}\right)$ where $E^{Ac}$, $E^{Al}$ and $E^{Ac,Al}$ are respectively the elastic energy of binding at the active site only (Ac), at the allosteric site only (Al) and at both sites simultaneously (Ac,Al). In the limit of weak elastic coupling between allosteric and active site, the fitness can be rewritten approximately as (see Sec. 1 in S1 Text)

$$\begin{array}{c} F\approx F^{Ac}\cdot R^{Al\to Ac} \end{array}$$ (1)

where ${\text{F}}^{Ac}$ is the force field imparted by substrate binding on the nodes of the active site, and $R^{Al\to Ac}$ is the displacement field induced at the active site by ligand binding. The product ${\text{F}}^{Ac}\cdot R^{Al\to Ac}$ is an estimate of the change of mechanical work required for binding the substrate at the active site caused by binding the ligand at the allosteric site. Note that each field in Eq 1 is of dimension *n*_0_ *d*, where *n*_0_ = 4 is the number of nodes in the active site and *d* = 2 the spatial dimension.

Such networks are evolved by changing the position of springs according to a Metropolis-Monte Carlo routine to maximize F. At each step, the fitness difference with respect to the previous configuration ΔF is computed and the new configuration is accepted with a probability p=min(1,expβΔF). *β* is an evolution inverse temperature controlling the selection pressure for high fitness F, we choose *β* = 10^4^ as at this temperature networks probed have the highest fitness our protocol can reach [30]. We sample every 1000 time steps after an initial equilibration time of 10^5^ steps. At long times one obtains a cooperative system of typical F∼0.2, whose architecture depends on the spatial dimension and boundary conditions [30]. Here we consider a network in *d* = 2 dimensions with periodic boundaries, equivalent to a cylindrical geometry, where the response to binding evolves towards a *shear* mode (see Fig 1A). With our scheme we can generate thousands of networks with a similar design. A sequence ***σ*** of 0 and 1, where *σ*_*i*_ = 1 stands for the presence of a spring at link *i* and *σ*_*i*_ = 0 for its absence, can be associated to any network, leading to a Multiple Sequence Alignment (MSA) of networks performing the same function (see Fig 1B).

## Results

### Nature and classification of epistasis

The cost of a single mutation (i.e. changing the occupancy) at some link *i* is defined as $\Delta F_{i}=F-F_{i}$ where F is the original fitness and $F_{i}$ the one of the network after the mutation. Single mutation costs $\Delta F_{i}$ are expected to be positive since the original network has been selected to have close-to-maximal fitness.

We denote by $\Delta F_{ij}=F-F_{ij}$ the cost of a double mutation at *i* and *j*. Epistasis between loci *i* and *j* is then defined as $\Delta \Delta F_{ij}\equiv \Delta F_{ij}-\Delta F_{i}-\Delta F_{j}$. We find that generically, the dominant effect of mutations is to affect the propagation of the signal $R^{Al\to Ac}$, which depends on the arrangement of links in the network. In general, mutations do not affect how binding at the active site locally generates force, as shown in Sec. 1 in S1 Text. Using this observation and following Eq 1, epistasis follows approximately

$$\begin{array}{c} \Delta \Delta F_{ij}\approx -F^{Ac}\cdot (\delta R_{ij}^{Al\to Ac}-\delta R_{i}^{Al\to Ac}-\delta R_{j}^{Al\to Ac}) \end{array}$$

where $\delta R_{i}^{Al\to Ac}=R_{i}^{Al\to Ac}-R^{Al\to Ac}$, and $R_{i}^{Al\to Ac}$ is the allosteric response at the active site of the protein mutated at link *i*. $\delta R_{j}^{Al\to Ac}$ and $\delta R_{ij}^{Al\to Ac}$ follow analogous definitions. We denote by *θ* the angle between $\delta R_{i}^{Al\to Ac}$ and $\delta R_{j}^{Al\to Ac}$.

Consider the case where the cost of a double mutation is dominated by the strongest point mutation, i.e. $\Delta F_{ij}\approx \text{max}\left(\Delta F_{i},\Delta F_{j}\right)$. It leads to:

$$\begin{array}{c} \Delta \Delta F_{ij}\approx -\text{min}\left(\Delta F_{i},\Delta F_{j}\right). \end{array}$$ (2)

**Fig 2:**
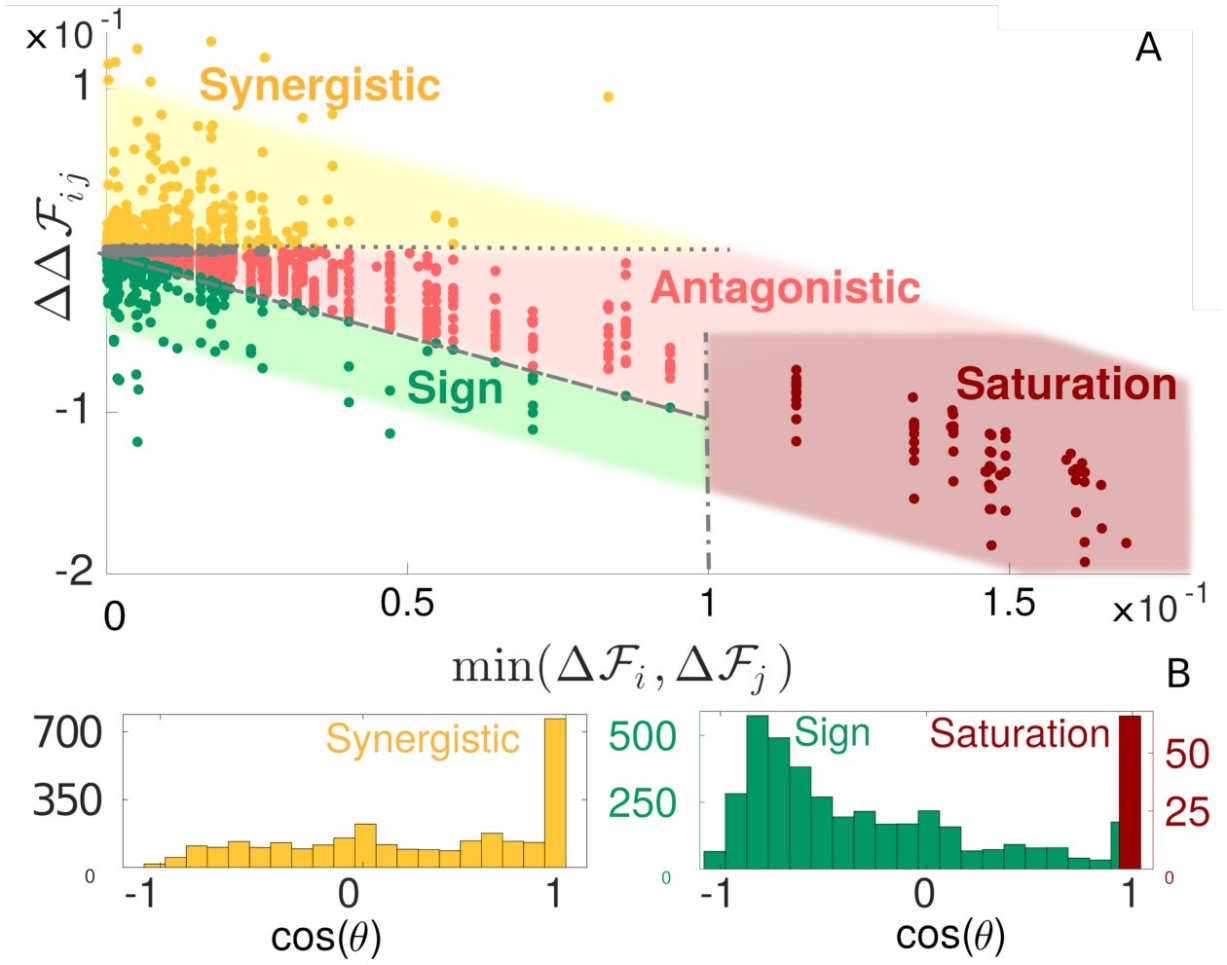
Classification and mechanical characterization of epistasis in our model of allosteric cooperativity. A: Phase diagram of epistasis in our allosteric material. All quantities are averages over 50 configurations obtained in a single run. The shaded area is taken with arbitrary width and a -1 slope as a guide to the eye. We show the lines $\Delta \Delta F_{ij}=0$ (dotted style), which corresponds to no epistasis (and divides synergistic from antagonistic/sign epistasis), $\Delta \Delta F_{ij}=\text{max}\left(\Delta F_{i},\Delta F_{j}\right)$ (dashed style), separating sign and antagonistic epistasis, and $\text{min}(\Delta F_{i},\Delta F_{j})=0.1$ (dash-dotted style), the threshold set to distinguish lethal mutations (corresponding to the saturation region). Points in grey correspond to epistasis < 5 × 10^−4^ and are excluded from our analysis. B: Histograms of *cos*(*θ*) for synergistic, sign and saturation epistasis.

Interestingly, this situation does capture the main trend of epistasis in our data, especially when it is strong, as shown in Fig 2A (see dashed line). This observation suggests to classify pairs of loci in terms of their epistasis and the minimal associated mutation cost $\text{min}(\Delta F_{i},\Delta F_{j})$ as performed in Fig 2A. First of all, no epistasis corresponds to purely additive mutations, i.e. $\Delta \Delta F_{ij}=0$, see dotted line in Fig 2A. Next, we observe the following regimes

*Saturation*: We define mutations with ΔF>0.1 as “lethal”. This somewhat arbitrary definition corresponds to 50% of loss of fitness. Pairs of such lethal mutations (which represent ∼ 0.1% of all pairs, a sparsity in line with experimental findings [24]) have the strongest epistasis in absolute value, and follow closely Eq 2, as visible in Fig 2A. Physically, these mutations essentially shut down signal propagation by themselves with $R_{i}^{Al\to Ac}\approx R_{j}^{Al\to Ac}\approx 0$, in such a way that the double mutation has the effect of a single one with $R_{ij}^{Al\to Ac}\approx 0$. This view is confirmed in Fig 2B by the observation that cos(*θ*) ≈ 1, as follows from $\delta R_{i}^{Al\to Ac}\approx \delta R_{j}^{Al\to Ac}\approx -R^{Al\to Ac}$. Saturation is then a form of very high “diminishing-returns” epistasis, for which evidence from data and support from theoretical models are accumulating [33, 34].
*Antagonistic*. Further up along the diagonal of Eq. 2 in Fig 2A, this saturation effect becomes milder. It is more akin to “antagonistic” epistasis [7, 35], whereby, after a first mutation, making a second one results only in a weak additional change. Antagonistic epistasis is also known as positive magnitude epistasis (where positivity indicates that the double mutant is fitter than expected from the additive case).
*Sign*. In the intermediate range of mutation costs with $\text{min}(\Delta F_{i},\Delta F_{j})<0.1$, more compensatory epistatic interactions can take place, where the fitness cost of a deleterious mutation is diminished by the second mutation (i.e. $\Delta F_{ij}<\text{max}\left(\Delta F_{i},\Delta F_{j}\right)$). Thus some mutations can become beneficial (i.e. increase the fitness) in presence of another mutation, and this resembles the “sign” epistasis empirically detected [7, 36]. Geometrically, it corresponds to situations where the two mutations deform the signal in opposite directions, so the second one can partially re-establish fitness. In support of this, Fig 2B shows that for sign epistasis cos(*θ*) tends to be negative.
*Synergistic*. Positive-sign values of $\Delta \Delta F_{ij}$ indicate “synergistic” epistasis. It occurs if two mutations perturb the elastic signal in the same direction, causing more damage than expected if they were purely additive. As clear from Fig 2B, cos(*θ*) tends to be positive in this case.

### Direct coupling analysis

We evolve numerically *M* configurations maximizing cooperativity F, each yielding a realization of a (variable) shear design. We sample a configuration for every initial condition to avoid introducing a bias in the sampling due to their high similarity. (We thus eliminate the possibility of our sequences to display “phylogenetic” effects, i.e. correlations due to a common evolutionary history, known to complicate the inference from sequence data and to require *ad hoc* corrections, see e.g. [37]). We find that the average Hamming distance among the obtained sequences is ∼ 20% of their length. Our set of sequences is analogous to a protein MSA—importantly, in this analogy the role of an amino-acid is played by a link, which can be stiff (*σ*_*i*_ = 1) or not (*σ*_*i*_ = 0, no springs). In practice we take *M* = 135000, much larger than the sequence length *N*_*c*_ = (3*L*^2^ − 2*L*) = 408. Working in such an over-sampling regime (which is generally not the case for real proteins) ensures that the limitations of the inference we find below are not due to sampling, but to the model underlying DCA.

**Fig 3:**
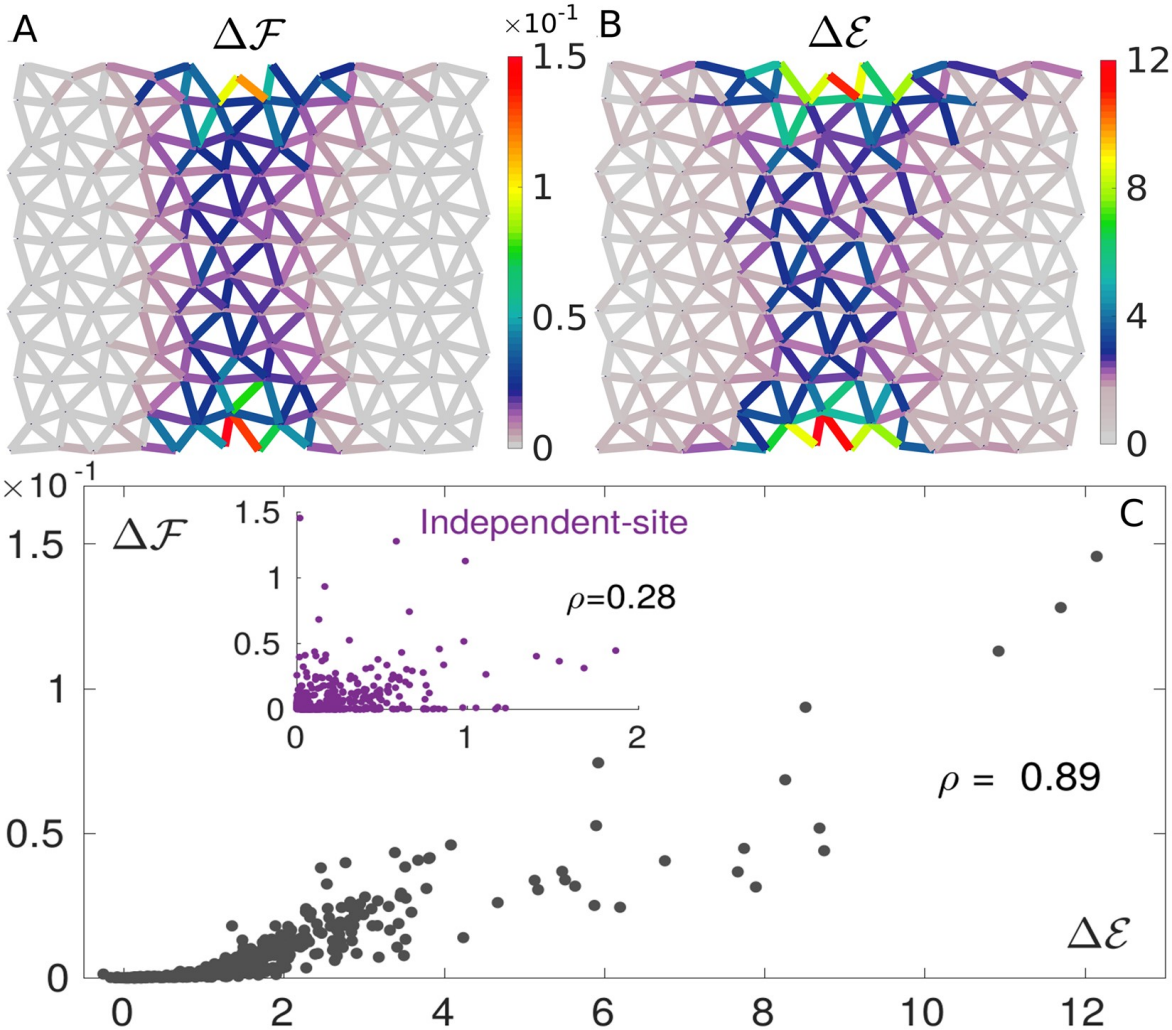
Prediction of mutation costs by DCA. Maps of true ΔF (A) and DCA-inferred ΔE (B) single mutation costs, averaged over 1.5 × 10^3^ configurations randomly chosen from the MSA. Their patterns are very similar, revealing high costs near the allosteric and active sites and in the shear path connecting them. C: Scatter plot showing the strong correlation between ΔF and ΔE for all links (averaged over 1.5 × 10^3^ configurations). The estimation of mutation costs based on an independent-site model (i.e. on conservation) correlates poorly with the true cost (inset), proving the need for incorporating correlations for proper prediction of mutation costs. The correlation is quantified via the Pearson correlation coefficient, *ρ*.

Next, for a statistical analysis of these sequences, we use DCA, which is based on the idea of fitting the observed single-site $\langle \sigma_{i}\rangle =1/M\sum_{m}\sigma_{i}^{m}$ and pairwise $\langle \sigma_{i}\sigma_{j}\rangle =1/M\sum_{m}\sigma_{i}^{m}\sigma_{j}^{m}$ frequencies of links by the probability distribution *P*(***σ***) with maximal entropy (as this ensures the least biased fit of data under such empirical constraints). In our setup this approach leads to

$$\begin{array}{ccc} P(\sigma ) & = & \frac{1}{Z}\exp\left(-E(\sigma )\right) \end{array}$$ (3)

$$\begin{array}{ccc} E(\sigma ) & = & -\sum_{i<j}J_{ij}\sigma_{i}\sigma_{j}-\sum_{i}h_{i}\sigma_{i} \end{array}$$ (4)

which is equivalent to an Ising model where *σ*_*i*_ = 0, 1 would denote the two states (down, up) of spins. In this setting, E is an estimation of βF, *β* being the inverse evolution temperature. In all the comparisons (e.g. Fig 3) we omit *β* as we are only interested in testing the proportionality between E and F. The “fields” *h*_*i*_ and “couplings” *J*_*ij*_ are inferred to match 〈*σ*_*i*_〉 and 〈*σ*_*i*_ *σ*_*j*_〉. The inference of these parameters can be performed with several algorithms, we focus on ACE (Adaptive Cluster Expansion) [38, 39], an approximate technique developed from statistical physics ideas, combined with maximum likelihood, an exact technique. This approach is extremely accurate and we compare it to a method more approximate, but much faster computationally, as mean field Direct Coupling Analysis (mfDCA) [16], see Methods for details on the implementation.

In this way we can benchmark DCA in the context of allosteric materials and test if it: (i) reproduces accurately the cost of single mutations; (ii) is a good generative model, i.e. if it can generate new sequences with high fitness and (iii) can predict epistasis.

#### Inferring mutation costs

Fig 3A shows the map of true mutation costs, indicating a large cost near the allosteric and active sites as well as in the central region where the allosteric response displays high shear (as documented in [30]). DCA enables one to infer this map by computing the estimated mutation cost $\Delta E_{i}=E_{i}-E$ for a mutation at a generic link *i*, Fig 3B. The comparison is excellent, as evident also from the high correlation revealed by the scatter plot Fig 3C. Importantly, including pairwise couplings is key for inferring mutation costs, as a model based on conservation alone (a standard measure of mutation costs, see Methods) performs poorly in this case, see inset of Fig 3C.

#### Generative power of DCA

**Fig 4:**
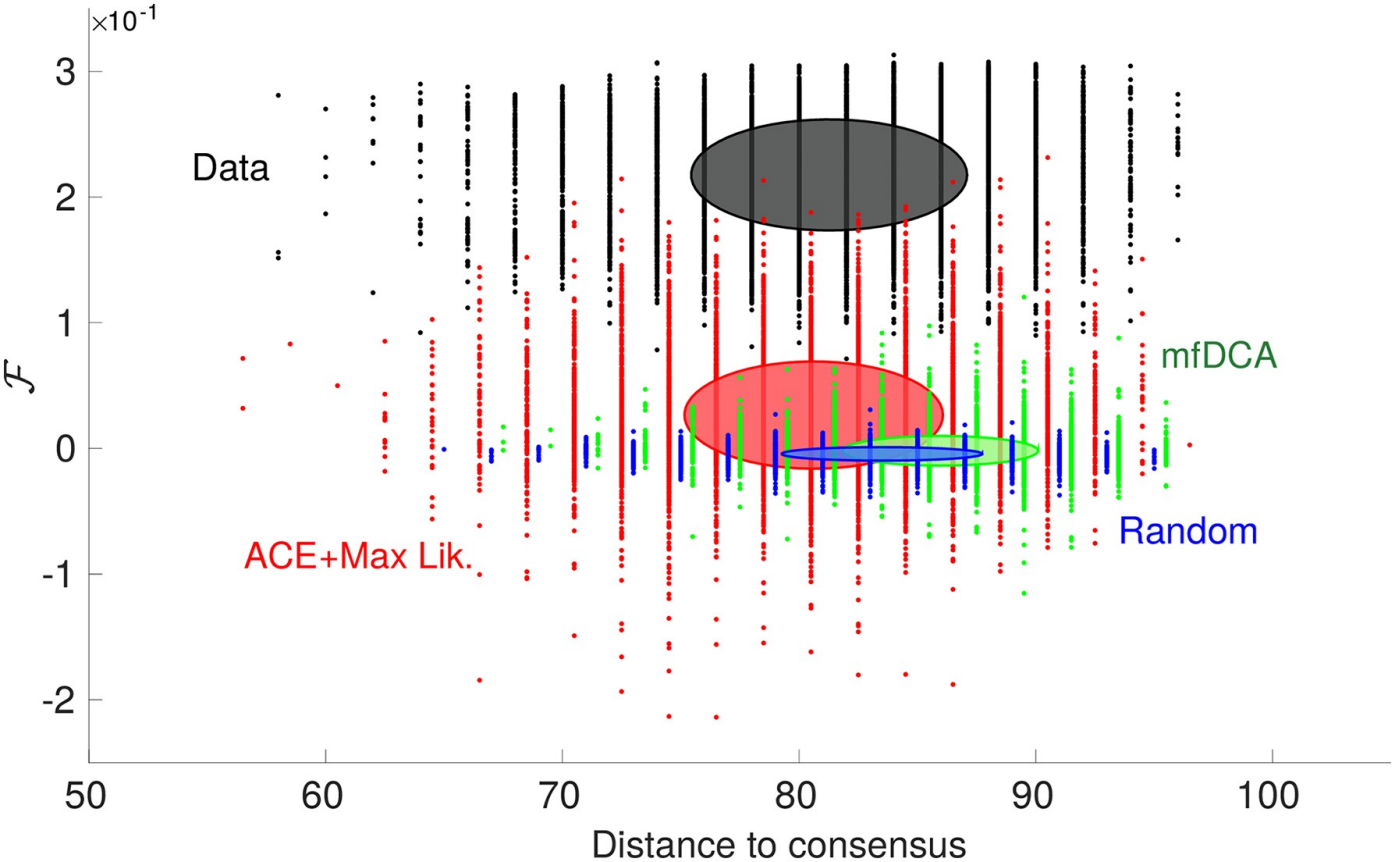
Generative performance of DCA. Fitness vs distance to consensus of configurations generated by the inferred model, following the representation of [40]. The sampling is done from *P*(***σ***) of Eq 4 (a Boltzmann-Gibbs probability distribution), whose parameters have been inferred via ACE + maximum likelihood (red cloud) or mfDCA (green cloud). Original high fitness configurations (black cloud) and random ones (blue) are added as a reference. Each cloud consists of 10^4^ sequences and the drawn ellipse gives one standard deviation around the mean in both horizontal and vertical directions. Distances to consensus of ACE + maximum likelihood, mfDCA and random sequences are shifted by respectively +0.7, −0.7 and −1.3 for better visibility.

Once the model of Eqs 3 and 4 is inferred, can it be used to generate new sequences with a high fitness, as previously shown for models of protein folding [40]? To answer this question, we generate new sequences by Monte Carlo sampling from the probability distribution Eq 3. Fig 4 shows the fitness of the obtained sequences vs their distance to “consensus”—the consensus being the most representative sequence of the MSA, i.e. where springs occupy the positions with largest mean occupancy. We find that (i) the variability of the MSA, quantified by the distance to consensus, is well reproduced (ii) the fitness is much more variable than for random sequences, with a few sequences that do perform as well as evolved ones (which never occurs for random sequences) but (iii) the mean obtained fitness is rather low, although larger, in a statistically significant way, than the one of random configurations (which is zero). As shown in Fig 4, these results deteriorate further if a more approximate algorithm as mfDCA is used to infer parameters. We have checked that the generative performance is not improved by lowering the temperature of the Monte Carlo sampling. Overall, these results suggest that the generative power of DCA is limited in the context of allostery, in contrast with results for models of protein folding [40]. Thus an Ising model, a quadratic model accounting for conservation and correlations in the MSA (first and second order statistics), although it can capture some features of the shear design (e.g. the inhomogeneous distribution of coordination, as shown in Fig. B in S1 Text), is a rather drastic approximation for the actual allosteric fitness. Indeed we have tested that higher orders as the third moment are not well reproduced (see Fig. A in S1 Text), suggesting that the longer-range correlations induced by allostery are not well captured by a pairwise model. On the other hand, for protein structure predictions, several works as [41] suggest that local correlations between residues in spatial contact are well-captured by a pairwise model, even beyond pairwise correlations. To test our findings, it would be interesting to condition the analysis of e.g. [41] on the distance between residues considered and see if the 3-body correlations are still captured when the residues are further apart. It would also be relevant to restrict the study to allosteric proteins only, to check whether statistical properties are changed, in such a way as to gauge the effect of allosteric vs folding constraints in proteins.

In what follows we shall emphasize in particular the failure of DCA to infer long-range epistasis.

### Inferring epistasis with DCA

**Fig 5:**
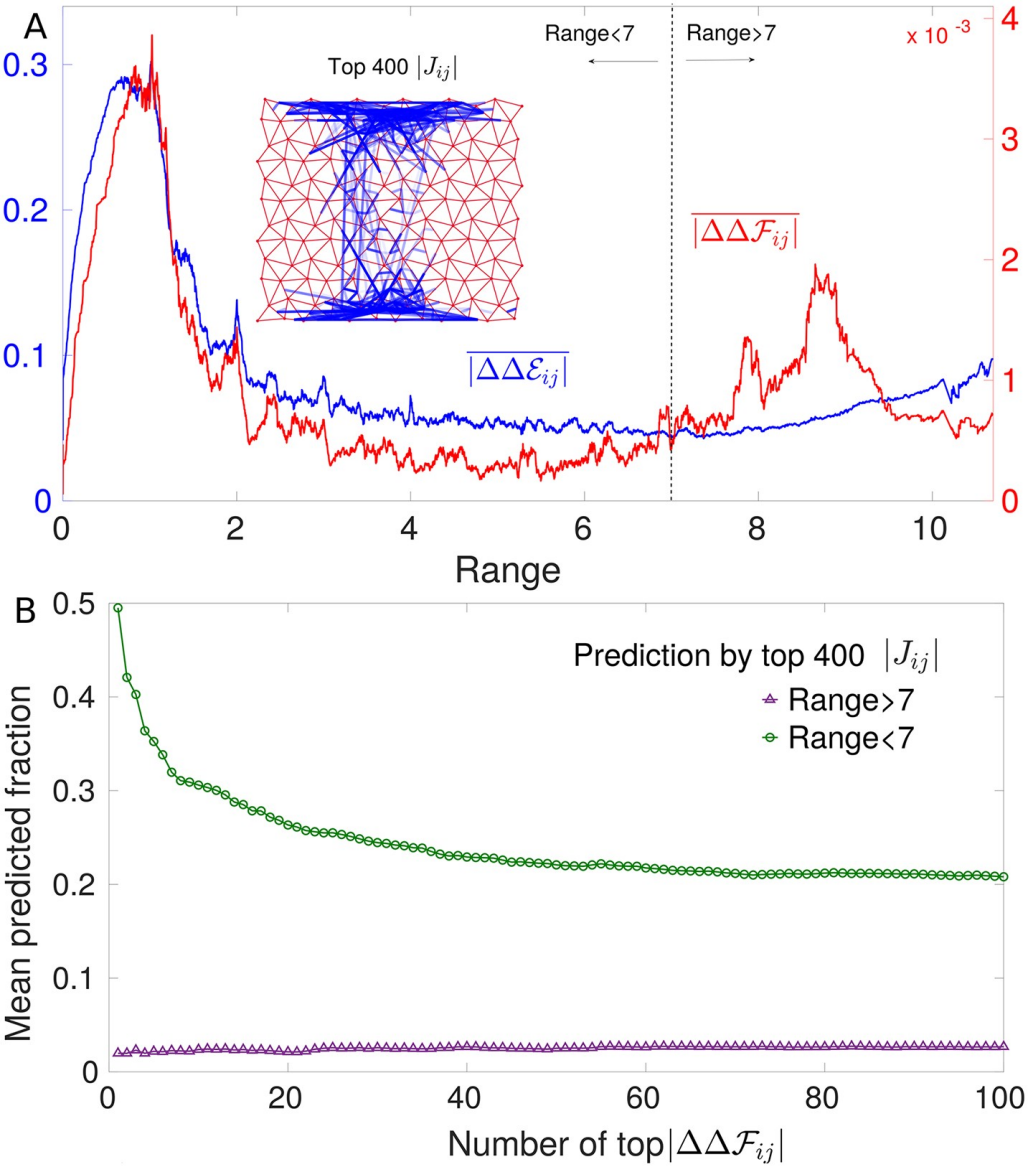
Prediction of epistasis by DCA. A: Running average of the absolute value of epistasis $\Delta \Delta F_{ij}$ and of DCA prediction $\Delta \Delta E_{ij}$ for 1.5 × 10^3^ configurations as a function of the distance between link *i* and *j*. The trends are nearly identical at short distances but at long distance DCA underestimates epistasis. Inset: Top 400 inferred couplings. They are mostly short range with only a few long-range couplings connecting the allosteric and the active site. Next we assess the prediction of epistasis in single configurations by these top 400 couplings. We consider separately long-range (> 7) and short-range (< 7) pairs of links, and rank them respectively in terms of the epistasis magnitude $|\Delta \Delta F_{ij}|$. B shows which fraction of these pairs—averaged over 100 configurations randomly chosen—belongs to the 400 largest couplings, as a function of the number of pairs with maximal epistasis considered. Clearly coupling magnitude has less predictive power at large distances than at short ones. The random expectations for these mean predicted fractions are 0.0041 for short-range pairs and 0.0009 for long-range ones (they are both significantly lower than the values reported here). This feature stays robust also if we increase, e.g. up to 1000, the number of top couplings for prediction (see Panel A in Fig. D, S1 Text).

From Eq 4 one readily has that the DCA prediction for epistasis follows $\Delta \Delta E_{ij}=-J_{ij}\left(2\sigma_{i}-1\right)\left(2\sigma_{j}-1\right)$, implying $|\Delta \Delta E_{ij}\left|=\right|J_{ij}|$. Hence, within DCA, the epistasis magnitude is simply the one of evolutionary couplings. In the inset of Fig 5A we show the spatial location of the top 400 pairs of links with highest coupling magnitude, illustrating that long-range couplings are rare. Yet, as implied jointly by Fig 2A (showing that pairs of sites with large mutation cost systematically display strong epistasis) and Fig 3A (showing that sites with a large mutation cost can be distant), long range epistasis is present in our model, meaning that DCA fails to capture it. This fact is demonstrated quantitatively in Fig 5A showing the mean epistasis $|\Delta \Delta F_{ij}|$ and mean DCA prediction $|\Delta \Delta E_{ij}|$ as a function of distances. The DCA-predicted trend reproduces the original one at small distances but strongly underestimates long-range epistasis. This is further evidenced in Fig 5B showing that the average fraction of long-range pairs (range > 7) with the largest epistasis which falls in the list of the 400 pairs with largest couplings is much smaller than for short-distance pairs (< 7). However, even at short distance the prediction by |*J*_*ij*_| is not excellent but it is remarkably improved if, as done in [12, 24], one considers epistasis averaged over several configurations (see Sec. 2 in S1 Text). (This result is in contrast to the remarkable performance of DCA in residue contact prediction, which guided the discovery of novel protein structures [17]. We recall that couplings inferred by the most accurate DCA algorithms exhibit maximal precision (i.e. number of true predicted contacts divided by the total number of predictions equal to 1) up to a number of contacts comparable with the protein size [42, 43]). Our finding is consistent with the lack of empirical evidence for long-range inferred couplings in allosteric proteins [25].

**Fig 6:**
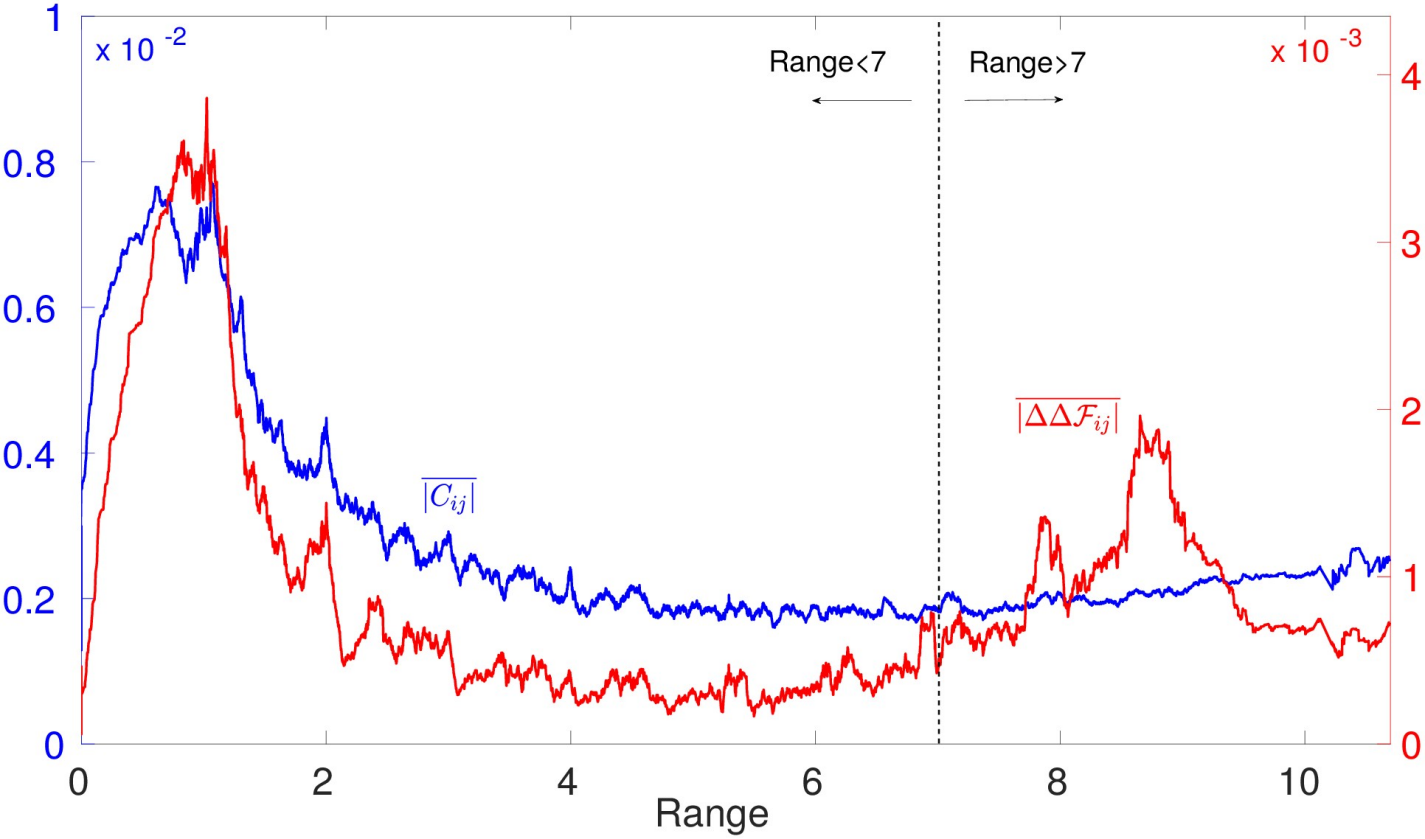
Running average of the absolute value of connected correlations *C*_*ij*_ = 〈*σ*_*i*_ *σ*_*j*_〉 − 〈*σ*_*i*_〉〈*σ*_*j*_〉 and of epistasis $\Delta \Delta F_{ij}$ for the same 1.5 × 10^3^ configurations of Fig 5A as a function of the distance between link *i* and *j*.

To better investigate the reasons for this phenomenon in our *in silico* model, we report evolutionary correlations as a function of distance in Fig 6. We find that, although strong long range epistasis occurs, large long-range correlations are absent (a fact in some sense more surprising that not finding long-range couplings, since in principle short-range couplings alone could result in long-range correlations). The absence of long-range correlations suggests that it will be particularly challenging to capture long-range functional dependencies from low order statistics of the MSA alone. Consistently with this observation, statistical approaches based on principal components of the MSA covariance such as Sectors [44, 45] or Inverse Covariance Off-Diagonal (ICOD) [46] do not lead overall to better predictions of epistasis in our context, as we show in S1 Text, Sec. 2.2. Among these approaches, we find that the best predictor of long-range epistasis is ICOD, a result that would be interesting to benchmark also in other systems.

#### A proposed explanation for the failure of DCA at long-distances

We propose that the failure of DCA at long-range stems from its inability to describe a function that requires many subparts of the system to work in concert, when each subpart can be of different type. For example, in allosteric proteins on short length scales soft regions must exist where shear propagates [30, 47], giving rise to local constraints. Yet, the exact location of these soft regions can vary in space. On a larger length scale, these regions must assemble to create an extended soft elastic mode [30, 48, 49], which generates global constraints: for the shear architectures it implies the presence of a soft path between the allosteric and active site, whose position however can fluctuate.

**Fig 7:**
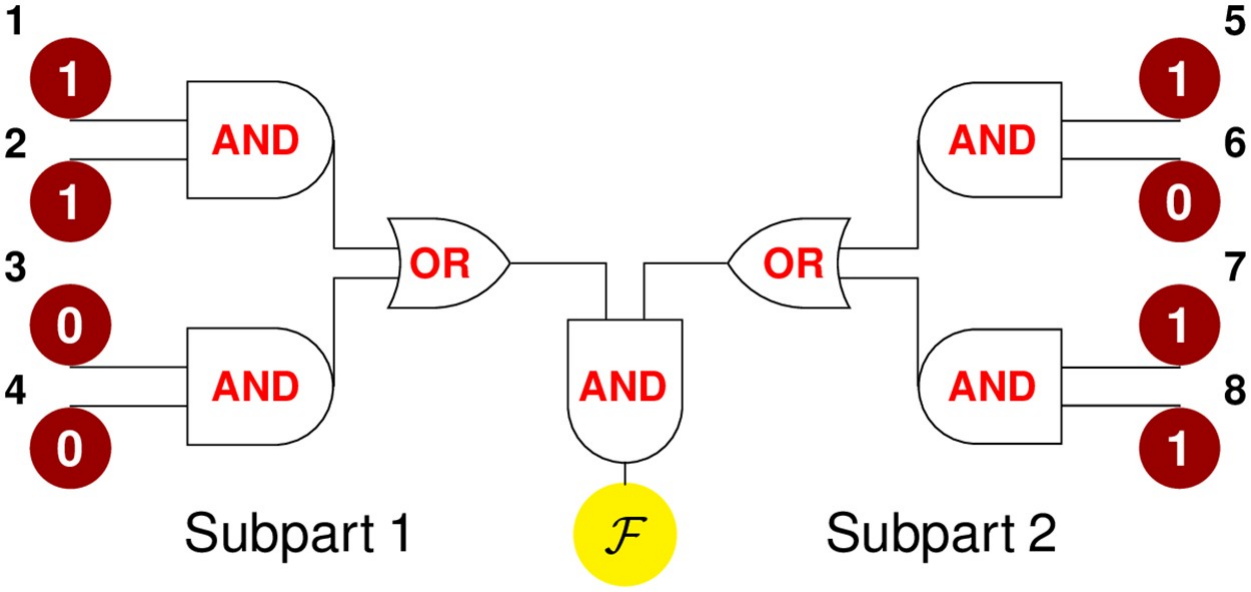
Sketch of a simple model for protein function. A system is arranged into 2 subparts which must work jointly to accomplish a given function (AND gate). Each subpart is composed of 2 groups, i.e. can be of 2 types (OR gate), to work each type must satisfy some constraints (AND gate between single units).

We argue that when applied to systems whose function is organized in such a hierarchical way, DCA underestimates long-range constraints. To illustrate this point, we introduce a Boolean model, shown in Fig 7. A generic “function” is achieved by two subparts that must work in concert (AND gate) and that can be of two different types (OR gate) but each must be functional (AND gate). This model comprises 8 units, taking the value 0 or 1, decomposed into 4 groups: 2 groups are the possible types of subpart 1 (left in Fig 7) and the other 2 the possible types of subpart 2 (right). A configuration is “functional” if 2 units of the same group are simultaneously in state 1 for each subpart. There are 49 functional configurations, whose fitness is fixed to F, all other configurations have fitness 0. We assume that F is large in such a way that the sequences in the MSA are only the 49 functional ones, with a uniform distribution. It is straightforward to calculate epistasis in this model, as well as single-site and pairwise frequencies from which couplings *J*_*ij*_ and fields *h*_*i*_ can be inferred. In particular we can compare $\Delta \Delta F_{ij}$ and $\Delta \Delta E_{ij}$ for units *i* and *j* either in the same group (or in the same subpart), so locally constrained by function (at “short distance”, e.g. *i* = 1 and *j* = 2), or in the two different subparts, thus globally constrained (at “long distance” e.g. *i* = 1 and *j* = 5). We obtain (see Sec. 2.1 in S1 Text) that $|\Delta \Delta F_{12}|/|\Delta \Delta F_{15}|\approx 2.3$: global and local constraints lead to relatively similar short range and long-range epistasis. Yet we find that epistasis between subparts is noticeably underestimated by DCA in contrast to epistasis within subparts. To show this, we look at the DCA prediction for the ratio of epistasis between two pairs of sites divided by the true ratio of epistasis. For pairs of sites belonging to the same subpart, DCA predicts equally well epistasis. For example, considering the pair of sites (1,2) and the pair (1,3), one finds $|\Delta \Delta E_{13}|/|\Delta \Delta E_{12}|\times |\Delta \Delta F_{12}|/|\Delta \Delta F_{13}|\approx 0.86$ which is close to unity. However if sites belong to different subparts, DCA strongly underestimates epistasis with $|\Delta \Delta E_{15}|/|\Delta \Delta E_{12}|\times |\Delta \Delta F_{12}|/|\Delta \Delta F_{15}|\approx 0.33$, i.e. by 3 fold. In this model as well we find that long-range correlations are essentially absent (they are smaller than 1%), despite long-range epistasis being present. Hence, a functional constraint on the cooperation between subparts potentially far away in the structure, as allosteric and active site, implies strong long-range epistasis, but does not imply strong long-range correlations, which is then reflected in small couplings. To summarize these facts, numerical values for correlation, epistasis and inferred couplings are listed in Table 1. Overall, this situation is precisely that of the *in silico* allosteric material (Figs 5 and 6), supporting that the present toy model captures the essence of the DCA limitations in more realistic settings.

**Table 1:** Table summarizing true and predicted epistasis magnitude, $|\Delta \Delta F_{ij}|$ and $|\Delta \Delta E_{ij}|$, connected correlations *C*_*ij*_ and inferred couplings *J*_*ij*_ in the simple model for sites *i* and *j* in the same group, in the same subpart and in different subparts. For *i* and *j* in different subparts (third row) the sizeable magnitude of epistasis is not reflected in the values of correlations, thus of the inferred couplings, in such a way that it is then underestimated by the DCA model. In section Sec. 2.1 in S1 Text, we derive $|\Delta \Delta F_{ij}|=21/49\;F$ for *i* and *j* in the same group: since we do not predict the prefactor F, we can fix 21/49F=1 and other numbers in the first column follow from this choice.

	$|\Delta \Delta F_{ij}|$	*C*_*ij*_	$|\Delta \Delta E_{ij}|$	*J*_*ij*_
Same group	1	0.061	0.51	1.18
Same subpart	0.33	−0.08	0.14	-1.01
Different subpart	0.43	0.00	0.07	0.40

#### Empirical evidence

**Fig 8:**
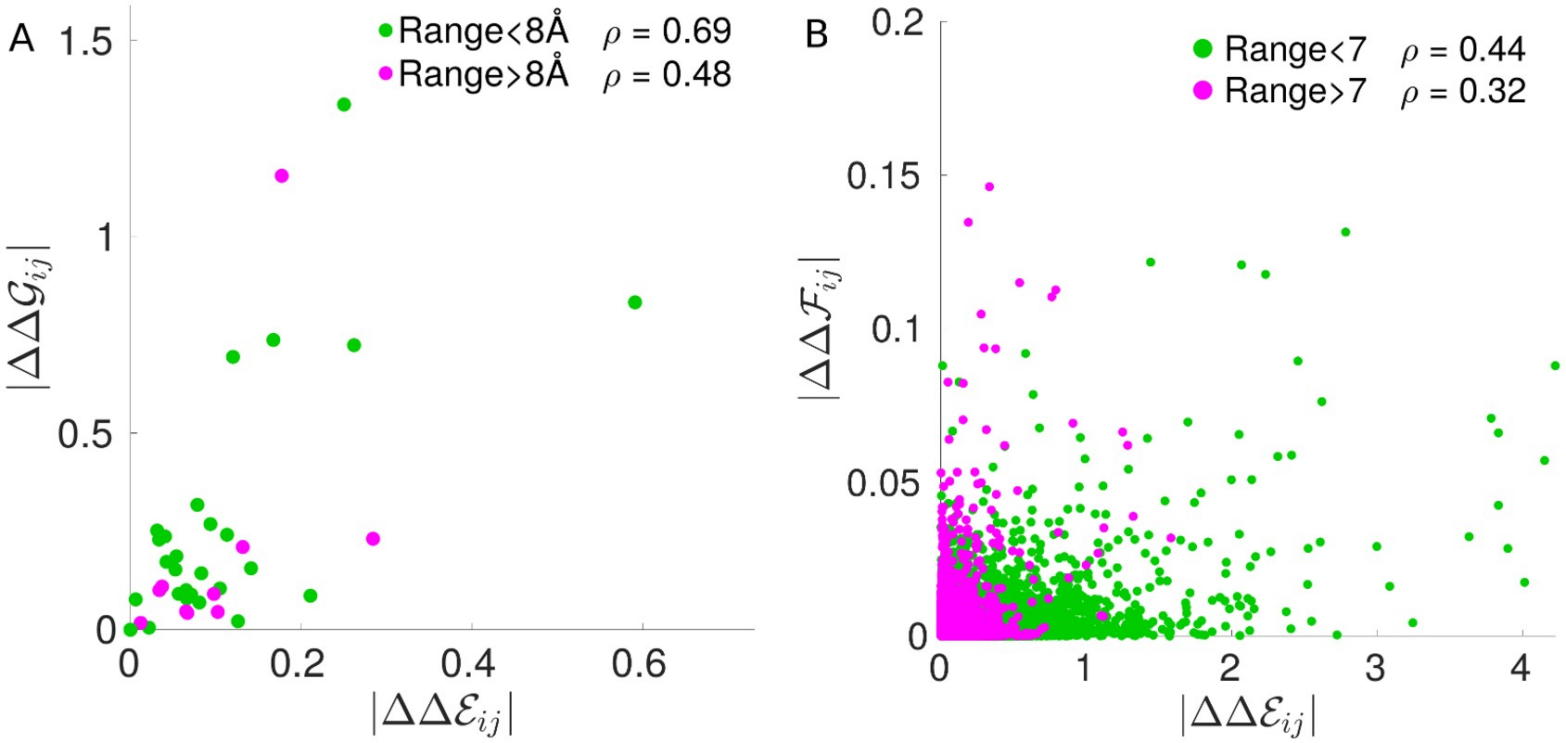
Prediction of experimentally measured epistasis by DCA from [12]. A: Scatter plot of average epistasis magnitude |ΔΔG| vs DCA-inferred energetic couplings |ΔΔE|, where the color code distinguishes short and long distance pairs of residues on the PDZ *α*2-helix three-dimensional structure. *ρ*, the Pearson correlation coefficient, indicates a better performance at short range. As a comparison, in B we show the scatter plot of average epistasis magnitude |ΔΔF| vs DCA-inferred energetic couplings |ΔΔE| in our *in silico* evolved networks: similarly to A, the prediction at long distance is poorer than at short distance.

Recently epistasis was measured in an empirical setting by Salinas and Ranganathan [12] with the aid of deep mutational scan techniques applied to the PDZ domain *α*2-helix (9 residues), which is part of an allosteric regulatory mechanism controlling ligand binding. Five homologs of PDZ domain were considered in the study. There, epistasis is

$$\begin{array}{c} \Delta \Delta G_{ij}^{xy}=(\Delta G_{i}^{x}+\Delta G_{j}^{y})-\Delta G_{ij}^{xy} \end{array}$$ (5)

where G is the binding free energy and *x*, *y* correspond to mutations happening at positions *i*, *j*, respectively. DCA inference in [12] was performed on an alignment of 1656 eukaryotic PDZ domains (Poole alignment, see [12]), from where the DCA epistasis prediction $|\Delta \Delta E_{ij}^{xy}|$ could be directly estimated. The authors then considered averages over mutations *x*, *y* and the 5 homologs (we denote them simply as $\Delta \Delta E_{ij}$ and $\Delta \Delta G_{ij}$); in Fig 8A we show how well $|\Delta \Delta E_{ij}|$ predict the experimental energetic couplings $|\Delta \Delta G_{ij}|$ for pairs of residues (*i*, *j*) at distance > 8Å and < 8Å, where distances are measured on the known three-dimensional crystal structure of the PDZ *α*2-helix and averaged over the 5 homologs. We find a stronger correlation between |ΔΔG| and |ΔΔE| for short range pairs (Pearson correlation *ρ* = 0.69), than for long range pairs (*ρ* = 0.48), as the long-range strong epistatic interaction between residues 1 and 8 is not captured by the DCA-inferred energetic couplings, see discussions in [12]. $|\Delta \Delta G_{18}|$ in Fig 8A is the point at largest |ΔΔG| in the long-range set. This observation is consistent with our model prediction, shown in Figs 5 and 8B, on the limits of DCA in capturing strong long-range epistasis.

It would be important to test more broadly this predicted effect, which may be possible thanks to the advances of deep mutational scans.

## Discussion

We have benchmarked DCA in a model of protein allostery where a mechanical task must be achieved over long distances. Such models display a rich pattern of epistasis, which can be both short and long-range and vary in sign. DCA predicts well mutation costs but is not a good generative model. This failure echoes with the drastic underestimation of long-range epistasis by the pairwise couplings inferred by DCA from evolutionary correlations. This finding rationalizes why there is no statistical evidence for long-range couplings in allosteric proteins analyzed by DCA [25], where long-range epistasis and functional effects are however found [6, 12, 15], as tested here with the data from [12].

Yet, as we show in S1 Text (see Sec. 2), we expect that DCA can capture some aspects of the long-range epistasis pattern in allosteric proteins. Indeed, high-cost mutations exhibit stronger epistasis than low-cost ones (as also seen in RNA sequences [36, 50], in the enzyme TEM-1 *β*-lactamase [11] and in previous *in silico* evolution work [32]), and are well-predicted by DCA. Specifically, the scaling of epistasis of Eq 2 suggests as approximation $|\Delta \Delta F_{ij}|\propto \text{min}\left(\Delta E_{i},\Delta E_{j}\right)$ where ΔE are inferred by DCA. Testing this prediction for epistasis patterns empirically could be made possible by the increasing availability of deep mutational scans [12, 51].

Moreover, we have provided the more general argument, illustrated by a simple model, that a co-evolution based maximum-entropy approach as DCA is not the appropriate inference framework when function requires several, variable parts to work in concert. Can one find better generative models than DCA for such complex functions? Several ways have been proposed to go beyond pairwise models by including nonlinearities, which implicitly take into account correlations at all orders, as nonlinear potentials in Restricted Boltzmann Machines [52], maximum-entropy probability measures with a nonlinear function of the energy [53], maximum-likelihood inference procedures based on nonlinear functions [54] and, finally, deeper architectures [55, 56]. As a first test, we have trained a 3-layers feedforward neural network with nonlinear (sigmoid) activation functions to learn the values of fitness in the simple model of Fig 7 and we have obtained that mutation costs and epistasis can be correctly captured by this method (see Sec. 2.1.1 in S1 Text). This observation raises the possibility that neural networks may lead to better generative models in proteins, a hypothesis that could also be benchmarked *in silico*.

Finally, as a future direction it would be interesting to extend our model by considering the constraint that the protein must fold to operate, in addition to the allosteric constraint considered here. It could be done for example in the spirit of [40] by considering that nodes are amino-acids, and that the stiffness of the spring between two adjacent amino-acids as well as their contribution to the total folding energy depend on the identity of that pair. Although we believe that such a model will lead to similar results as presented here for long-range coupling, it will presumably differ significantly in the statistics of short range ones. In particular, it may capture why 3-body correlations are well described by 2-body correlations in real proteins, and lead to stronger conservation overall [55].

## Methods

### Direct coupling analysis: Inference procedure

In a maximum-entropy approach, extracting information from MSAs can be cast as an inverse problem, i.e. inferring the set of parameters which enable the model (an Ising model in our setup) to reproduce certain observed statistical properties [57, 58]. The exact solution of this problem is found by Maximum Likelihood algorithms, which search for the set of couplings *J*_*ij*_ and fields *h*_*i*_ maximizing the likelihood that the model specified by such parameters produced data with the given statistics (single-site and pairwise frequencies in our case). This exact maximization might often be infeasible, therefore to tackle the inverse problem approximate techniques have been developed: for instance, we resort to the Adaptive Cluster Expansion (ACE), an expansion of the entropy (which indeed corresponds to the likelihood) into contributions from clusters of spins [38, 39, 42]. We use the package made available by Barton https://github.com/johnbarton/ACE. The implementation consists of first a run of ACE followed by a proper maximum likelihood refinement (QLS routine), which takes as starting set of fields and couplings the ACE-inferred ones. Different parameters for the ACE and QLS routines can be set by the user, e.g. *γ*_2_, the *L*_2_–norm regularization strength for couplings which penalizes spurious large absolute values induced by undersampling and for which a natural value is *γ*_2_ = 1/*M* (*M* being the size of the sample). To help convergence, we have chosen for ACE a higher value *γ*_2_ = 10^−2^ and *θ* = 10^−5^ (this is the threshold at which the algorithm will run then exit, see [39]). In the further refinement by QLS, we have set *mcb*, the number of Monte Carlo steps used to estimate the inference error, to 200000 and *γ*_2_ = 1/*M*. Having full control of the numerical evolution, we have tried to avoid undersampling issues by generating a large number of configurations *M* = 135000, which leads to *γ*_2_ ≈ 0.7 × 10^−5^. For the inference we remove from sequences the 6 links at the active and allosteric sites as they are always associated to the symbol 1 (always occupied by a spring), so the number of parameters to infer is $N_{c}^{\prime }+N_{c}^{\prime }\left(N_{c}^{\prime }-1\right)/2∼81000$ with $N_{c}^{\prime }=N_{c}-6=402$. We have verified that low values of the *L*_2_-regularization allow us to obtain the maximal generative performance compatible with the model (in comparison to higher regularization). By default the *L*_2_ regularization of fields is 0.01 × *γ*_2_. In Panel A in Fig. A of S1 Text, it is shown that the result of the inference is a model perfectly able to reproduce the first and second order statistics (as it should by construction) but that fails at reproducing higher order statistics.

For a comparison, we have considered also mean field Direct Coupling Analysis (mfDCA) [16], derived from a mean-field factorized ansatz for the Boltzmann-Gibbs distribution Eq 3. Couplings in mfDCA are given by *J*_*ij*_ = −(***C***^−1^)_*ij*_, where ***C***_*ij*_ = 〈*σ*_*i*_ *σ*_*j*_〉 − 〈*σ*_*i*_〉〈*σ*_*j*_〉 is the covariance of the MSA (we recall that in each sequence *σ*_*i*_ = 1 stands for the presence of a spring at link *i* and *σ*_*i*_ = 0 for its absence). Typically ***C*** is not invertible due to undersampling, making it necessary to add a pseudocount λ (see [37]). As shown in [59], a pseudocount also helps correct for the systematic biases introduced by the mean field approximation: for this reason, we have used a pseudocount λ and chosen its value as λ = 0.5, which allows the best comparison to the ACE and maximum likelihood results, see Panel B in Fig. A of S1 Text. It is noteworthy that in this way a computationally cheap technique as mfDCA yields a pattern of top *J*_*ij*_ strikingly similar to the one of a very accurate inference achieved by the combination of ACE and maximum likelihood. Therefore mfDCA, while extremely poor as a generative model, exhibits a good performance at reconstructing the distribution of relevant couplings, as shown in Panel C, Fig. A in S1 Text.

### Mutation costs and generative performance in the inferred Ising model

Costs of double mutations, i.e. joint mutations affecting links *i* and *j*, can be computed in the original model via fitness changes $\Delta F_{ij}=F-F_{ij}$, where $F_{ij}$ is the fitness after springs in *i* and *j* have been mutated. A double mutation can correspond either to (i) adding two springs at links *i* and *j* (i.e. *σ*_*i*_ = *σ*_*j*_ = 1) or removing them (i.e. *σ*_*i*_ = *σ*_*j*_ = 0) or to (ii) moving a spring from link *i* to link *j* or viceversa (i.e. *σ*_*i*_ = 0, *σ*_*j*_ = 1 or *σ*_*i*_ = 1, *σ*_*j*_ = 0). Let us call the former “non-swap” mutations and the latter “swap” mutations. Swap mutations conserve the total amount of springs (360), thus the overall average coordination 〈*z*〉 = 5, and are the ones performed in the *in silico* evolution. As optimal allosteric configurations maximize fitness with respect to this type of mutations, we stick to them also when we compare mutation costs in terms of fitness and inferred energy (see Fig 3C): we define “effective” single mutation costs $\Delta F_{i}$ and $\Delta E_{i}$ by taking, for each link, the swap with a link in the external region (more rigid, as visible in e.g. Fig. B of S1 Text), where mutations are completely neutral, thus whose cost would be roughly zero.

For the generative step, we implement a Monte Carlo sampling which relocates springs from an occupied to an unoccupied link, i.e. which follows swap-type dynamics as for the original numerical evolution. This allows us to select, from the inferred model, sequences that are structurally as close as possible to the initial data, i.e. with the same average coordination 〈*z*〉 = 5, to make a consistent comparison with them. We have verified that even relaxing this constraint in the sampling leads to sequences endowed with higher internal variability yet lying in the same range on fitness (hence the inferred model incorporates rather well the information on the fixed amount of springs). The parameters of the Ising model are inferred in such a way as to match single-site occupancy, which reflects the spatial pattern of coordination in the allosteric networks. In Fig. B of S1 Text we show that generated sequences, despite having lower fitness, reproduce successfully this property as they should.

#### Comparison with conservation

Single-site frequency in protein alignments, informative about local conservation, is a standard measure of mutation costs at a certain position [60] and can be fit by an independent-site Ising model. Energy (Eq 4) in this case contains only field terms and, once these are inferred from link occupancies 〈*σ*_*i*_〉, one can compute energy changes $\Delta E_{i}$ upon point mutations. The energy cost of a mutation in an independent-site model is then $\Delta E_{i}=\left(2\sigma_{i}-1\right)h_{i}$, where $h_{i}=\text{log}\left(\langle \sigma_{i}\rangle \left(1-\bar{\sigma }\right)/\bar{\sigma }\left(1-\langle \sigma_{i}\rangle \right)\right)$ describes how the observed occupancy of a link *i*, 〈*σ*_*i*_〉, is biased away from the average occupancy $\bar{\sigma }=360/408=0.88$. In average $\Delta E_{i}$ gives also a measure of *conservation* of link *i* as it is 0 when $\langle \sigma_{i}\rangle =\bar{\sigma }$ and it increases the more link *i* tends to be either occupied or vacant. The improvement achieved by the pairwise model over this conservation-based measure of mutation costs is extremely significant (see inset of Fig 3C). On the one hand, conservation is a purely local measure—it takes into account how a particular position is crucial to the propagation of the allosteric response. Including pairwise couplings proves to be crucial to capture the context-dependence of mutation costs, and thus must be included for their quantitative prediction. On the other hand, the degree itself of structural conservation is rather low due to the heterogeneity of the shear-design MSA: the conformation, precise location and size of the shear path, hence the role of each link, can vary from architecture to architecture, leading to low structural conservation (with peaks only around the active and allosteric site). Conservation is found much higher *within* one set of dynamically related solutions (as for Fig 2A), corresponding to one realization of the shear design among the many included in the MSA (see in particular Fig. 4G in [30]).

## Supporting information

S1 Text Supporting information for “Direct coupling analysis of epistasis in allosteric materials”. (PDF)
