# Supplementary material for: Direct coupling analysis of epistasis in allosteric materials
Source: PLoS Comput Biol. 2020 Mar 2;16(3):e1007630. doi: 10.1371/journal.pcbi.1007630 (PMC7067494; doi:10.1371/journal.pcbi.1007630)
Supplement: S1 Text — (PDF) [file pcbi.1007630.s001.pdf]

Supplementary Information S1 Text:

Direct Coupling Analysis of Epistasis in Allosteric Materials

Barbara Bravi[1], Riccardo Ravasio[1], Carolina Brito[2], Matthieu Wyart[1]

[1] *Institute of Physics, École Polytechnique Fédérale de Lausanne, CH-1015 Lausanne, Switzerland*

[2] *Instituto de Fìsica, Universidade Federal do Rio Grande do Sul, CP 15051, 91501-970 Porto Alegre RS, Brazil*

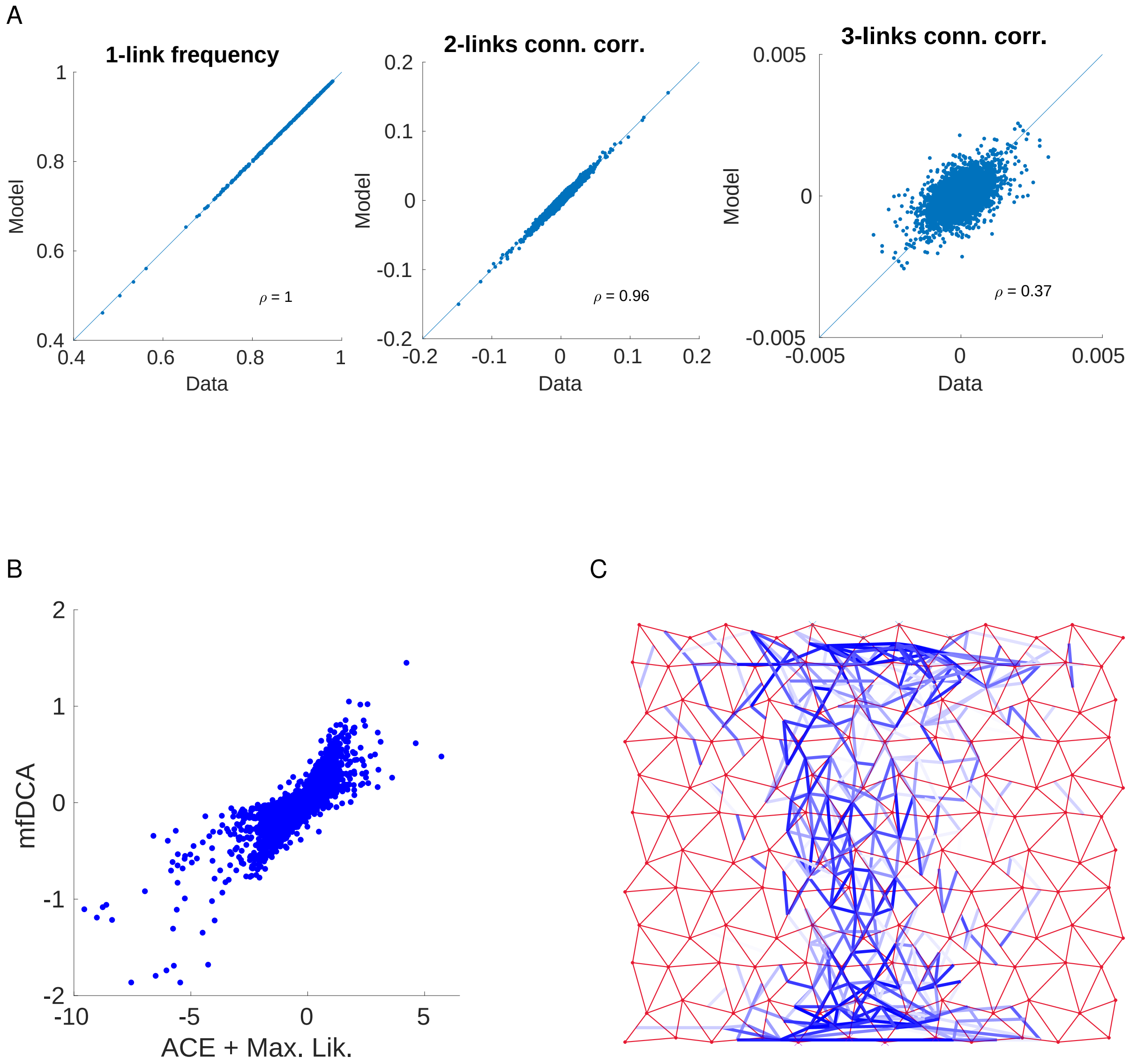

Figure A: **Performance of the inference procedure**. A: Statistics of the model inferred by combining ACE and Maximum Likelihood. 1-link frequency and 2-links connected correlations are very accurately reproduced, as they should by construction (the relative errors, defined as in [39], are respectively $\epsilon_m = 2.45 \times 10^{-1}$ and $\epsilon_C = 1.30 \times 10^{-1}$). In contrast the third order connected correlations, which are not constrained in the inference, are not well captured (Pearson correlation coefficient $\rho = 0.37$). This is a hint that the Ising model - a pairwise probabilistic model over σ_i - is an approximation which becomes poor for estimating higher order moments. B: Scatter plot comparing J_{ij} inferred via mfDCA to the direct couplings of ACE + Max. Lik.: the pseudocount in mfDCA has been set to $\lambda = 0.5$ in such a way as to obtain the highest correlation between the two. C: Spatial distribution of top 400 mfDCA-inferred couplings on the network. The reconstruction of the topology of relevant couplings is rather robust with respect to the choice of more approximate inference methods as mfDCA. As in Fig. 5A (inset) of the main text, they are concentrated at short range, i.e. they connect links lying close either to the active site or the allosteric site and in the central high-shear path. Long range mfDCA couplings, connecting links around respectively allosteric and active site, are weaker and appear among the top 600-1000 ones, implying an even worse performance at predicting long range epistasis than ACE + Max. Lik.

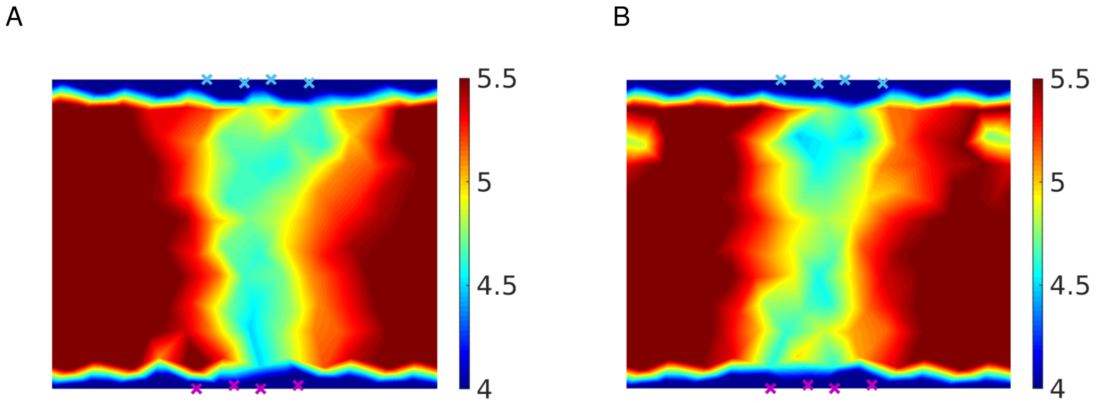

Figure B: **Properties of generated allosteric sequences**. Coordination map of original sequences (A) and generated ones (B). They both exhibit a softer (i.e. with coordination $z < 5$) central path joining active and allosteric sites (indicated respectively by blue and purple crosses) along which the shear-like sliding takes place. This path is embedded in a more connected, "rigid" region where the coordination $z > 5$. Solutions sampled from the inferred energy landscape have the expected design but are not maximally fit, showing that more "structural" components, as the distribution of links, are captured but additional information would be needed to reproduce a complex mechanical function as the cooperative fitness.

1 Mechanical interpretation of mutation costs and epistasis

Let us denote by ϵ the set of nodes where ligand binding takes place, e.g. for ligand binding at the allosteric site $\epsilon = (\mathcal{A}l)$ with size $\dim(\epsilon) = n_0$. Such event imposes a displacement $\mathbf{R}^\epsilon$ on the nodes ϵ which imparts locally a force $\boldsymbol{F}^\epsilon$ and induces a response $\mathbf{R}^{\epsilon\to r}$ on all the other nodes r. Clearly $\dim(\epsilon) + \dim(r) = L^d$ where L^d is the total number of nodes for a network of size L in d dimensions; for the example of binding to the allosteric site $r = (\mathcal{A}c, b)$, where b stands for the "bulk" of nodes belonging neither to the allosteric nor to the active site. (In this paper we consider networks as in Fig. 1A of the main text, with $d = 2$, $L = 12$ and $n_0 = 4$ for both active and allosteric site). Considering the deformation as a linear response to the external force, the relation between force and overall response field is written in terms of the dynamical matrix $\boldsymbol{\mathcal{M}}$

$$\begin{pmatrix} \boldsymbol{F}^\epsilon \\ \mathbb{0} \end{pmatrix} = \boldsymbol{\mathcal{M}} \begin{pmatrix} \mathbf{R}^\epsilon \\ \mathbf{R}^{\epsilon\to r} \end{pmatrix} \tag{6}$$

hence $\boldsymbol{\mathcal{M}}$ is endowed with a block structure as follows

$$\boldsymbol{\mathcal{M}} = \begin{pmatrix} \boldsymbol{\mathcal{M}}^{\epsilon,\epsilon} & \boldsymbol{\mathcal{M}}^{\epsilon,r} \\ (\boldsymbol{\mathcal{M}}^{\epsilon,r})^T & \boldsymbol{\mathcal{M}}^{r,r} \end{pmatrix}$$

For pairwise interactions such as springs, $\boldsymbol{\mathcal{M}}$ is symmetric. Forces as well as responses can be calculated solely from the imposed displacement by introducing a matrix $\boldsymbol{\mathcal{Q}}$

$$\boldsymbol{\mathcal{Q}} = \begin{pmatrix} \mathbb{1}^\epsilon & -\boldsymbol{\mathcal{M}}^{\epsilon,r} \\ \mathbb{0} & -\boldsymbol{\mathcal{M}}^{r,r} \end{pmatrix}$$

such that

$$\begin{pmatrix} \boldsymbol{F}^\epsilon \\ \mathbf{R}^{\epsilon\to r} \end{pmatrix} = \boldsymbol{\mathcal{Q}}^{-1} \boldsymbol{\mathcal{M}} \begin{pmatrix} \mathbf{R}^r \\ \mathbb{0} \end{pmatrix} \tag{7}$$

Binding at ϵ costs an elastic energy E^ϵ

$$E^\epsilon = \frac{1}{2} \boldsymbol{F}^\epsilon \cdot \boldsymbol{R}^\epsilon \tag{8}$$

and the cooperative fitness is defined by a combination of such elastic energies

$$\mathcal{F} = E^{\mathcal{A}c} - (E^{\mathcal{A}c,\mathcal{A}l} - E^{\mathcal{A}l}) \tag{9}$$

where $E^{\mathcal{A}c}$, $E^{\mathcal{A}c,\mathcal{A}l}$ and $E^{\mathcal{A}l}$ are given by Eq. 8 with $\epsilon = (\mathcal{A}c)$, $\epsilon = (\mathcal{A}c, \mathcal{A}l)$ and $\epsilon = (\mathcal{A}l)$ respectively. Maximal cooperativity corresponds to making binding of a substrate at the active site energetically favored when already a ligand is bound to the allosteric site, as this reduces its binding energy from $E^{\mathcal{A}c}$ to $(E^{\mathcal{A}c,\mathcal{A}l} - E^{\mathcal{A}l})$. One can express the energy of joint binding at the allosteric and active site $E^{\mathcal{A}c,\mathcal{A}l} = \frac{1}{2}\boldsymbol{F}^{\mathcal{A}c,\mathcal{A}l} \cdot \boldsymbol{R}^{\mathcal{A}c,\mathcal{A}l}$ as

$$\frac{1}{2} \boldsymbol{F}^{\mathcal{A}c,\mathcal{A}l} \cdot \boldsymbol{R}^{\mathcal{A}c,\mathcal{A}l} = \frac{1}{2} \boldsymbol{F}^{\mathcal{A}l} \cdot \boldsymbol{R}^{\mathcal{A}l} + \frac{1}{2} \boldsymbol{F}^{\mathcal{A}c}_{|\mathcal{A}l} \cdot (\boldsymbol{R}^{\mathcal{A}c} - \boldsymbol{R}^{\mathcal{A}l\to\mathcal{A}c}) \tag{10}$$

i.e. after binding at the allosteric site with an energy cost $\frac{1}{2}\boldsymbol{F}^{\mathcal{A}l} \cdot \boldsymbol{R}^{\mathcal{A}l}$, the elastic energy of binding at the active site is determined by (i) the force there when a ligand is already bound at the allosteric site

($\boldsymbol{F}^{\mathcal{A}c}_{|\mathcal{A}l}$ with subindex $|\mathcal{A}l$); (ii) the displacement imposed at the active site $\boldsymbol{R}^{\mathcal{A}c}$ to which we subtract the response already caused by ligand binding at the allosteric site $\boldsymbol{R}^{\mathcal{A}l\to\mathcal{A}c}$. Eq. 10 allows us to rewrite Eq. 9 as

$$\mathcal{F} = \frac{1}{2}\,\boldsymbol{F}^{\mathcal{A}c}_{|\mathcal{A}l}\cdot\boldsymbol{R}^{\mathcal{A}l\to\mathcal{A}c} + \frac{1}{2}\,\delta\boldsymbol{F}^{\mathcal{A}l\to\mathcal{A}c}\cdot\boldsymbol{R}^{\mathcal{A}c} \tag{11}$$

where one has $\boldsymbol{F}^{\mathcal{A}c} - \boldsymbol{F}^{\mathcal{A}c}_{|\mathcal{A}l} = \delta\boldsymbol{F}^{\mathcal{A}l\to\mathcal{A}c}$.

We now consider the weak elastic coupling limit between the allosteric and active sites. Physically, we assume that the response induced at the active site by binding at the allosteric site is small compared to the one induced by binding at the active site. Mathematically, it corresponds to the assumptions that the elements $\boldsymbol{\mathcal{M}}^{\mathcal{A}c,b}(\boldsymbol{\mathcal{M}}^{b,b})^{-1}\boldsymbol{\mathcal{M}}^{b,\mathcal{A}l}$ are small. In this limit, expressing $\delta\boldsymbol{F}^{\mathcal{A}l\to\mathcal{A}c}$ and $\boldsymbol{R}^{\mathcal{A}l\to\mathcal{A}c}$ in terms of the imposed displacements by using Eq. 7, we find that each term in Eq. 11 follows, at first order in the $\boldsymbol{\mathcal{M}}^{\mathcal{A}c,b}(\boldsymbol{\mathcal{M}}^{b,b})^{-1}\boldsymbol{\mathcal{M}}^{b,\mathcal{A}l}$:

$$\frac{1}{2}\,\boldsymbol{F}^{\mathcal{A}c}_{|\mathcal{A}l}\cdot\boldsymbol{R}^{\mathcal{A}l\to\mathcal{A}c} \approx \frac{1}{2}\,\delta\boldsymbol{F}^{\mathcal{A}l\to\mathcal{A}c}\cdot\boldsymbol{R}^{\mathcal{A}c} \approx \frac{1}{2}\,(\boldsymbol{R}^{\mathcal{A}c})^T\cdot(\boldsymbol{\mathcal{M}}^{\mathcal{A}c,b})(\boldsymbol{\mathcal{M}}^{b,b})^{-1}(\boldsymbol{\mathcal{M}}^{b,\mathcal{A}l})\cdot\boldsymbol{R}^{\mathcal{A}l} \tag{12}$$

where a sum over b, the ensemble of "bulk" nodes, is taken. Hence, by using that $\frac{1}{2}\,\delta\boldsymbol{F}^{\mathcal{A}l\to\mathcal{A}c}\cdot\boldsymbol{R}^{\mathcal{A}c} \approx \frac{1}{2}\,\boldsymbol{F}^{\mathcal{A}c}_{|\mathcal{A}l}\cdot\boldsymbol{R}^{\mathcal{A}l\to\mathcal{A}c}$, we obtain from Eq. 11

$$\mathcal{F} \approx \mathbf{F}^{\mathcal{A}c}\cdot\boldsymbol{R}^{\mathcal{A}l\to\mathcal{A}c} \tag{13}$$

since $\boldsymbol{F}^{\mathcal{A}c}_{|\mathcal{A}l}$ can be approximated by $\boldsymbol{F}^{\mathcal{A}c}$ in the weak coupling limit.

If we denote by $\mathbf{F}^{\mathcal{A}c}_i$ and $\boldsymbol{R}^{\mathcal{A}l\to\mathcal{A}c}_i$ forces and displacements after a mutation at link i, the cost of one mutation can be expressed in this approximation (see Panel B Fig. C for a numerical validation of our approximation) as $\Delta\mathcal{F}_i \approx \Delta(\mathbf{F}^{\mathcal{A}c}\cdot\boldsymbol{R}^{\mathcal{A}l\to\mathcal{A}c})_i$, where $\Delta(\mathbf{F}^{\mathcal{A}c}\cdot\boldsymbol{R}^{\mathcal{A}l\to\mathcal{A}c})_i = \mathbf{F}^{\mathcal{A}c}\cdot\boldsymbol{R}^{\mathcal{A}l\to\mathcal{A}c} - \mathbf{F}^{\mathcal{A}c}_i\cdot\boldsymbol{R}^{\mathcal{A}l\to\mathcal{A}c}_i$. It can be further rewritten as

$$\Delta(\mathbf{F}^{\mathcal{A}c}\cdot\boldsymbol{R}^{\mathcal{A}l\to\mathcal{A}c})_i \approx -\big(\mathbf{F}^{\mathcal{A}c}\cdot\delta\boldsymbol{R}^{\mathcal{A}l\to\mathcal{A}c}_i + \delta\mathbf{F}^{\mathcal{A}c}_i\cdot\boldsymbol{R}^{\mathcal{A}l\to\mathcal{A}c} + \delta\mathbf{F}^{\mathcal{A}c}_i\cdot\delta\boldsymbol{R}^{\mathcal{A}l\to\mathcal{A}c}_i\big) \tag{14}$$

having defined changes in force as $\delta\mathbf{F}^{\mathcal{A}c}_i = \mathbf{F}^{\mathcal{A}c}_i - \mathbf{F}^{\mathcal{A}c}$ in analogy to changes in displacement $\delta\boldsymbol{R}^{\mathcal{A}l\to\mathcal{A}c}_i$ introduced in the main text. We find numerically that the cost of single mutations, when it is not too small, is dominated by the changes in displacement at the active site

$$\Delta\mathcal{F}_i \approx -\mathbf{F}^{\mathcal{A}c}\cdot\delta\boldsymbol{R}^{\mathcal{A}l\to\mathcal{A}c}_i \tag{15}$$

as implied jointly by in Panels B and C Fig. C. As a consequence, epistasis between mutations at i and j with significant magnitude can be written $\Delta\Delta\mathcal{F}_{ij} \approx -\mathbf{F}^{\mathcal{A}c}\cdot\big(\delta\boldsymbol{R}^{\mathcal{A}l\to\mathcal{A}c}_{ij} - \delta\boldsymbol{R}^{\mathcal{A}l\to\mathcal{A}c}_i - \delta\boldsymbol{R}^{\mathcal{A}l\to\mathcal{A}c}_j\big)$, as presented in the main text. Displacement vectors and their changes upon high-cost mutations at the active site are schematically depicted in Panel A Fig. C.

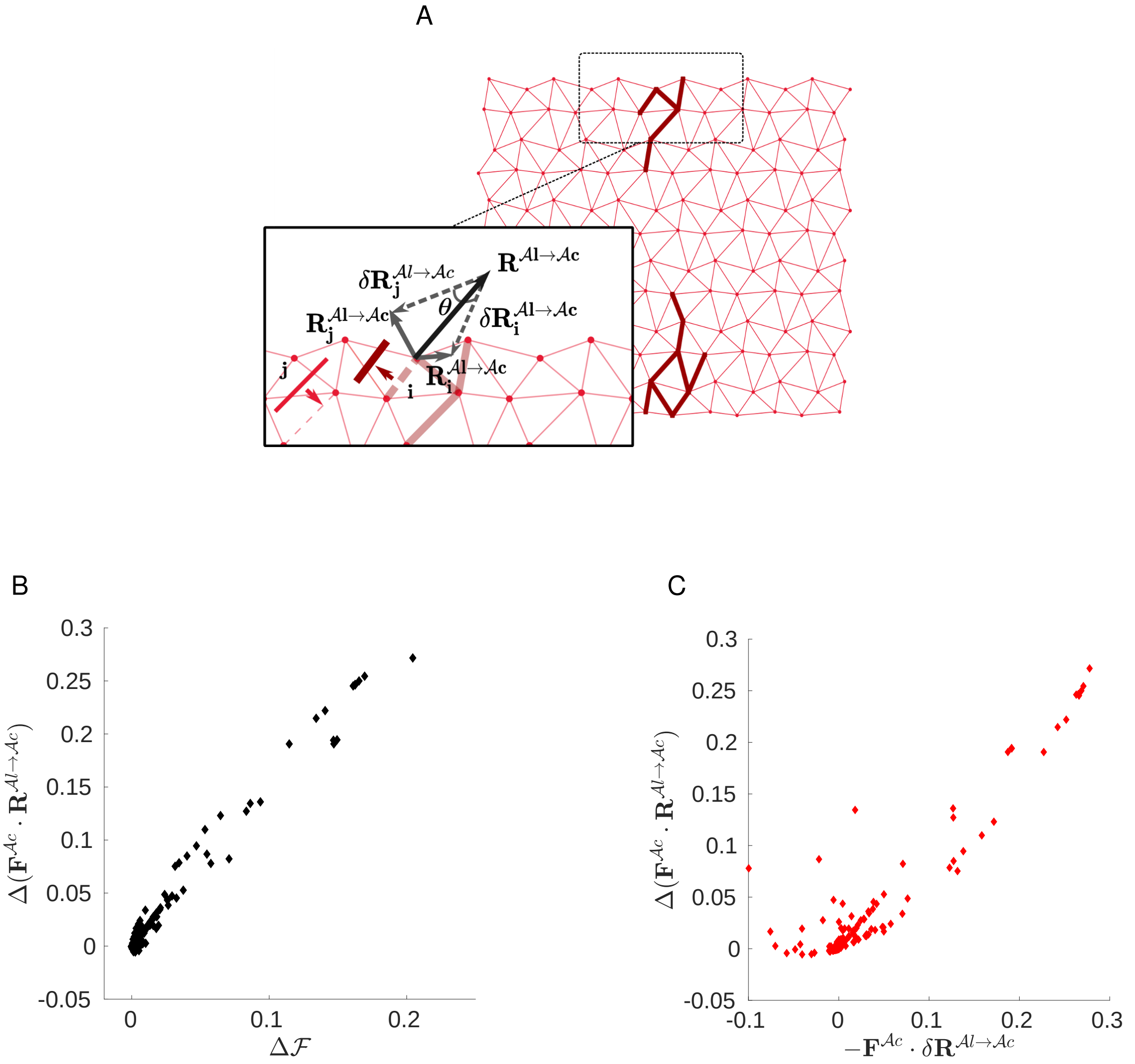

Figure C: **Mechanics of mutations**. A: The geometry of mutation costs is illustrated in the zoom on the active site region (note that for simplicity of visualization we consider only one of the $n_0 = 4$ nodes). Thick, dark red lines highlight links whose disruption would be lethal for the allosteric fitness. These few links, crucial to the long-distance propagation of the allosteric response, are located around active and allosteric site and exhibit maximal epistasis along with maximal single mutation costs (i.e. they populate the saturation region of Fig. 2A in the main text). After a lethal mutation consisting in removing a spring at link i, the displacement at the active site $\mathbf{R}_i^{\mathcal{A}l\to\mathcal{A}c}$ is significantly reduced with respect to the original optimal displacement $\mathbf{R}^{\mathcal{A}l\to\mathcal{A}c}$ and their difference is given by $\delta\mathbf{R}_i^{\mathcal{A}l\to\mathcal{A}c}$ (dashed arrow). When a second lethal mutation at j occurs, we denote by θ the angle between $\delta\mathbf{R}_i^{\mathcal{A}l\to\mathcal{A}c}$ and $\delta\mathbf{R}_j^{\mathcal{A}l\to\mathcal{A}c}$; for lethal mutations $\cos(\theta) \approx 1$ (see Fig. 2B in the main text), i.e. they all tend to have a homogeneous direction of action which is precisely the one opposite to the displacement at the active site. B and C: Numerical test of the approximation $\Delta\mathcal{F}_i \approx \Delta(\mathbf{F}^{\mathcal{A}c} \cdot \boldsymbol{R}^{\mathcal{A}l\to\mathcal{A}c})_i$ (B) and of $\Delta(\mathbf{F}^{\mathcal{A}c} \cdot \boldsymbol{R}^{\mathcal{A}l\to\mathcal{A}c})_i \approx -\mathbf{F}^{\mathcal{A}c} \cdot \delta\boldsymbol{R}_i^{\mathcal{A}l\to\mathcal{A}c}$ (C). The latter is valid only for medium-high mutation costs.

2 Prediction of epistasis

The scaling of epistasis (Eq. 2 in the main text) suggests a measure simply based on the inferred single mutation costs, i.e. $|\Delta\Delta\mathcal{F}_{ij}| \propto \min(\Delta\mathcal{E}_i, \Delta\mathcal{E}_j)$ with $\Delta\mathcal{E}$ inferred by DCA. We have verified that this procedure improves extremely the prediction of long-range epistasis in our model for allostery in comparison to the direct evolutionary couplings J_{ij}, both for single configurations and for the average epistatic pattern, as shown in respectively in Panels B and C Fig. D.

2.1 Simple model illustrating the failure of DCA

To explain the discrepancy between short-range and long-range DCA-predictions of epistasis, we resort to the simple model of Fig. 7 (main text). We assign to all the 49 functional configurations the same fitness $\mathcal{F}$, all the other $2^8 - 49$ configurations would not belong to the sample of optimal configurations and are taken with zero fitness, thus $\Delta\mathcal{F} = 0$ if a mutation (single or double) results in a configuration still belonging to the optimal sample and $\Delta\mathcal{F} = \mathcal{F}$ otherwise. If we model each unit as a spin $\sigma = 0, 1$, this fitness function can be mathematically written as

$$\mathcal{F} = \mathcal{F}(\sigma_1\sigma_2 + \sigma_3\sigma_4 - \sigma_1\sigma_2\sigma_3\sigma_4) \cdot (\sigma_5\sigma_6 + \sigma_7\sigma_8 - \sigma_5\sigma_6\sigma_7\sigma_8) \tag{16}$$

i.e. it introduces high order couplings both at short (within groups and subparts) and long range (across subparts).

We can estimate average mutation costs by counting how frequently mutations would lead to a configuration outside of the optimal sample, yielding

$$\Delta\Delta\mathcal{F}_{12} = \Delta\mathcal{F}_{12} - \Delta\mathcal{F}_1 - \Delta\mathcal{F}_2 = 21/49\mathcal{F} - 21/49\mathcal{F} - 21/49\mathcal{F} = -21/49\mathcal{F} \tag{17}$$

$$\Delta\Delta\mathcal{F}_{15} = 33/49\mathcal{F} - 21/49\mathcal{F} - 21/49\mathcal{F} = -9/49\mathcal{F} \tag{18}$$

$$\frac{|\Delta\Delta\mathcal{F}_{12}|}{|\Delta\Delta\mathcal{F}_{15}|} = 21/9 \approx 2.3 \tag{19}$$

Next, by a simple likelihood maximization we infer the set of J_{ij} and h_i compatible with $\langle\sigma_i\rangle$ and $\langle\sigma_i\sigma_j\rangle$, single-site and pairwise frequencies of the optimal sample. We estimate $J_{12} = 1.18$ and $J_{15} = 0.40$, thus the prediction by DCA

$$\frac{|\Delta\Delta\mathcal{E}_{12}|}{|\Delta\Delta\mathcal{E}_{15}|} = \frac{|J_{12}(2\langle\sigma_1\rangle + 2\langle\sigma_2\rangle - 4\langle\sigma_1\sigma_2\rangle - 1)|}{|J_{15}(2\langle\sigma_1\rangle + 2\langle\sigma_5\rangle - 4\langle\sigma_1\sigma_5\rangle - 1)|} = \frac{|J_{12}(-21/49)|}{|J_{15}(-9/49)|} \approx 6.9 \tag{20}$$

i.e. the DCA prediction is significantly biased towards short-range epistasis. Due to symmetry of our model, epistasis and the DCA-prediction for any combination of units in the two subparts is the same as for units 1 and 5; similarly, the result for 2 units within the same group is given by the values for units 1 and 2. For the remaining combinations of units, i.e. the ones belonging the same subpart but to different groups (e.g. $i = 1$ and $j = 3$) we obtain that epistasis is weaker compared to units within the same group

$$\frac{|\Delta\Delta\mathcal{F}_{12}|}{|\Delta\Delta\mathcal{F}_{13}|} = \frac{|-21/49\mathcal{F}|}{|-7/49\mathcal{F}|} = 3 \tag{21}$$

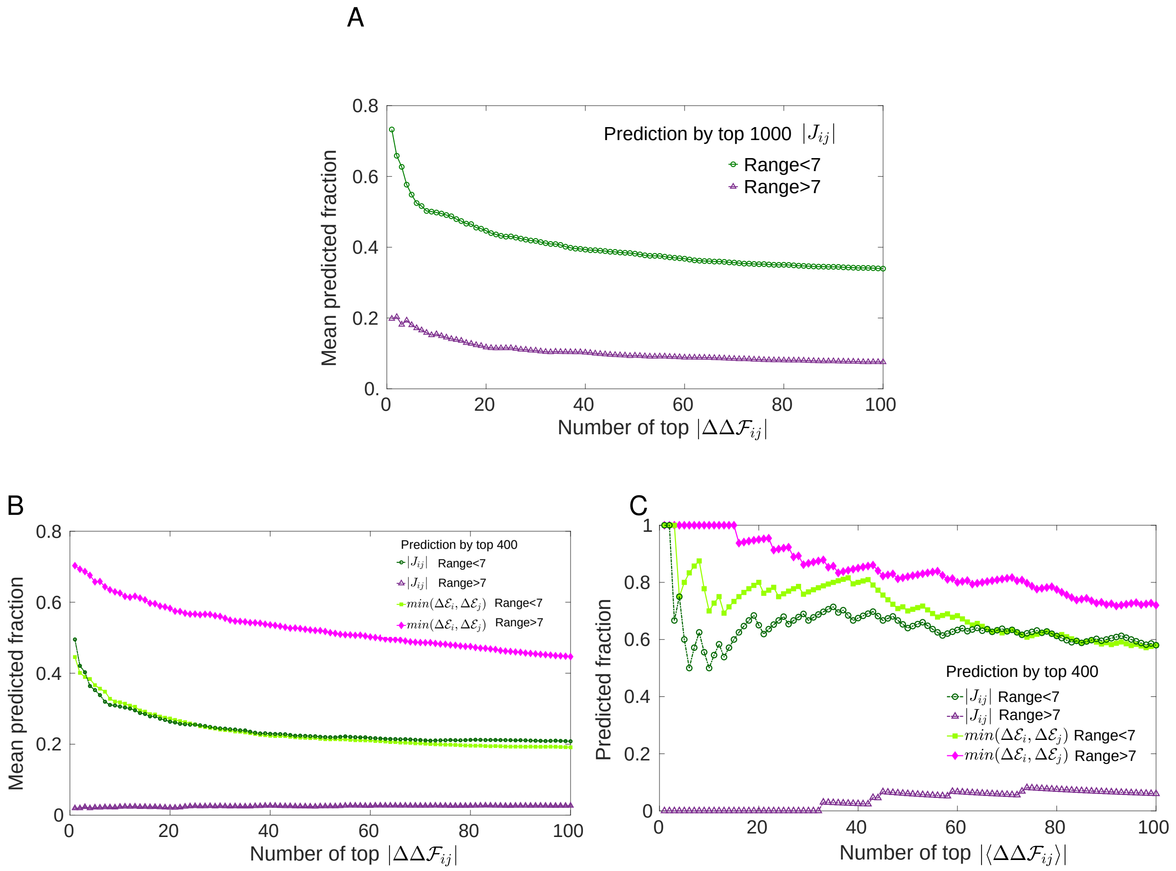

Figure D: **Prediction of epistasis by the DCA-inferred model**. A: Same plot as in Fig. 5B (main text) where we show the fraction of top rank epistasis $|\Delta\Delta\mathcal{F}_{ij}|$ predicted by top 1000 $|J_{ij}|$, averaged over 100 configurations. In comparison to Fig. 5B, here we consider a higher number of the couplings with largest magnitude to predict epistasis: the mean predicted fraction increases both for short range and long range epistasis, yet a clear difference between their values remains. B: Same plot as Fig. 5B (main text) where we added curves for the prediction by $\min(\Delta\mathcal{E}_i, \Delta\mathcal{E}_j)$ - the minimum between average single mutation costs at i and j - corresponding to scaling 2 in the main text, which describes well the trend of epistasis (see Panel A Fig. 2). As in Fig. 5B (main text), we rank separately long-range (> 7) and short-range (< 7) pairs of links i and j in terms of $|\Delta\Delta\mathcal{F}_{ij}|$ and we plot the fraction of these pairs - averaged over 100 configurations randomly chosen - falling either into the top 400 $|J_{ij}|$ (empty symbols) or into the top 400 values of $\min(\Delta\mathcal{E}_i, \Delta\mathcal{E}_j)$ (filled symbols). This second measure improves only slightly the estimation of strong short-range epistasis but it does so dramatically for long-range one. C: Same plot as B where we show the fraction of the average epistasis $\langle\Delta\Delta\mathcal{F}_{ij}\rangle$ (estimated from 1.5×10^3 randomly chosen configurations of the MSA) that one would predict either via $|J_{ij}|$ or $\min(\Delta\mathcal{E}_i, \Delta\mathcal{E}_j)$. The prediction at short distance is rather accurate, with the predicted fraction reaching 1 for the maximally epistatic pairs; at long distance, signal on long-range epistasis captured by $|J_{ij}|$ is almost absent while the prediction by $\min(\Delta\mathcal{E}_i, \Delta\mathcal{E}_j)$ stands out for its precision.

Since each subpart can be of different type (OR gate), units from different groups (i.e. types) are less tightly constrained by function. The DCA-prediction does not underestimate epistasis as for units of different subparts (i.e. at long distance) with

$$\frac{|\Delta\Delta\mathcal{E}_{12}|}{|\Delta\Delta\mathcal{E}_{13}|} = \frac{|J_{12}(-21/49)|}{|J_{13}(7/49)|} \approx 3.5 \tag{22}$$

where $J_{13} = -1.01$. From Eq. 19, Eq. 20, Eq. 21 and Eq. 22 it is straightforward to calculate $|\Delta\Delta\mathcal{E}_{13}|/|\Delta\Delta\mathcal{E}_{12}| \times |\Delta\Delta\mathcal{F}_{12}|/|\Delta\Delta\mathcal{F}_{13}| \approx 0.86$ and $|\Delta\Delta\mathcal{E}_{15}|/|\Delta\Delta\mathcal{E}_{12}| \times |\Delta\Delta\mathcal{F}_{12}|/|\Delta\Delta\mathcal{F}_{15}| \approx 0.33$.

2.1.1 Feedforward neural network

To understand which machine learning tools could improve the prediction of epistasis in the simple model, we have built a feedforward neural network performing least squares regression of sequence data based on their fitness (see Fig. E). For data in the training set, we provide the network with both the input sequence and the target answer, i.e. a label 1 (standing for fitness $\mathcal{F}$) or 0. We vary the size of the training set from 10% to 80% of the $2^8 = 256$ total sequences and we keep the remaining sequences of the sample for validation of the accuracy of prediction. We learn the weights, i.e. the connections between layers, which minimize the mean squared error between the output of the network and the target answers by stochastic gradient descent from a random orthogonal initialization. The 10% of learning runs with the best performance on the training dataset reach an average training error ranging between $\sim 4 \times 10^{-8}$ for a training set with 10% of the sample (25 configurations) to $\sim 3 \times 10^{-10}$ with 80%; the average validation error for the same runs is between $\sim 3 \times 10^{-1}$ and $\sim 2 \times 10^{-2}$ respectively. We repeated the learning with an architecture where the width of the first hidden layer is bigger than the length of input data, for instance 16 and 32. For a width of 16 hidden units, the top 10% of trainings maintains an average accuracy on the training set of order 10^{-8} for the smaller training set (10% of the sample) and of order 10^{-10} for the largest one (80% of the sample); the corresponding average validation errors are $\sim 3 \times 10^{-1}$ and $\sim 4 \times 10^{-2}$. When increasing further the first layer to a width of 32, we also added a dropout (here equal to 0.3) to balance the increase of parameters to learn with the same amount of data and avoid overfitting. In this way we obtained that the training error, averaged over the 10% best runs, was higher (from $\sim 8 \times 10^{-5}$ for a training set with 10% of the sample to $\sim 6 \times 10^{-6}$ with 80%) but the performance on the validation set was better (respective average errors of $\sim 2 \times 10^{-1}$ and 10^{-4}). Provided that the training set is not too small, these numerical tests confirm that a trained neural network, when presented with an optimal sequence mutated at some position, can predict the value of its fitness with good accuracy in such a way as to predict $\Delta\mathcal{F} \sim 0$ when it still belongs to the optimal sample or $\Delta\mathcal{F} \sim 1$ if it does not. This ensures that also epistasis would be accurately predicted at any range.

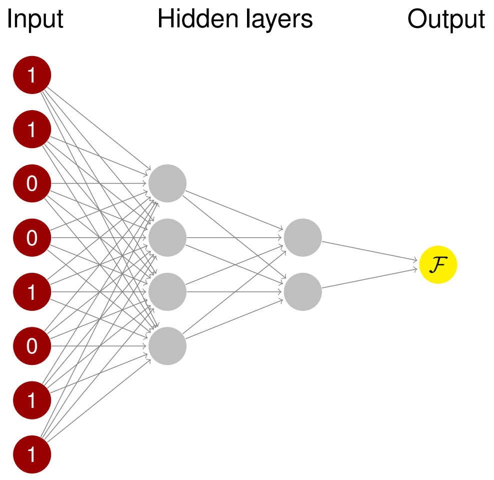

Figure E: **Graphical representation of the feedforward neural network for regression in the simple model**. The size of the input layer is 8, as the size of the system. We add two hidden layers of 4 and 2 units and the final one-unit output is 1 if the input sequence has fitness $\mathcal{F}$ and 0 otherwise. The activation function from one layer to the successive one is a sigmoid and the weights are dense (all units in one layer are connected to all units of the successive one).

2.2 Inferring epistasis by Statistical Coupling Analysis

Statistical Coupling Analysis (SCA) is a principal component analysis on the covariance matrix of MSA weighted by position conservation that allows one to select the so-called "Sectors" [44,45]. Basically, sectors consist of delocalized modes that chain together strongly co-evolving amino acids usually contiguous in the tertiary structure; they can be interpreted as basic evolutionary units that could underlie several functions including allostery [14, 15]. It has been recently shown [12] that SCA, and in particular the first principal component only, can correctly capture a larger portion of epistasis than DCA in a deep mutational scanning experiment on the PDZ domain. Spurred by this result, we take the covariance matrix between links in our MSA of artificially evolved networks

$$C_{ij} = \frac{1}{M}\sum_{m=1}^{M}\sigma_i^m\sigma_j^m - \left(\frac{1}{M}\sum_{m=1}^{M}\sigma_i^m\right)\left(\frac{1}{M}\sum_{m=1}^{M}\sigma_j^m\right) \tag{23}$$

We apply the conservation weight prescribed by sector analysis [45], i.e. we consider the principal components of the matrix $\phi_i\phi_j C_{ij}$ where ϕ_i is a correction weighting conservation at each site. ϕ_i is defined as $\phi_i = \partial D_i/\partial\langle\sigma_i\rangle$ where

$$D_i = \langle\sigma_i\rangle \ln\frac{\langle\sigma_i\rangle}{\bar{\sigma}} + \big(1-\langle\sigma_i\rangle\big)\ln\frac{1-\langle\sigma_i\rangle}{1-\bar{\sigma}} \tag{24}$$

is the divergence of the observed occupancy of link i from the background occupancy $\bar{\sigma}$ (see Methods) and is a measure of conservation at site i. Panel A Fig. F shows the spectrum of eigenvalues $\boldsymbol{\lambda}$ of $\phi_i\phi_j C_{ij}$: the top eigenvalue λ^1 is clearly separated from the bulk (which would be shared with the spectrum of a random sample) thus incorporates information on the functional features. As in [12], we reconstruct the covariance from the top eigenmode only, $\tilde{C}^1_{ij} = \lambda^1 v^1_i v^1_j$, where $\boldsymbol{v}^1$ is the eigenvector corresponding to λ^1

and its structure is visualized on the network in Panel B Fig. F. In Panel C Fig. F we plot the absolute value of $\tilde{C}^1_{ij}$ against epistasis magnitude: it does not improve the prediction of epistasis compared to the inferred $\Delta\Delta\mathcal{E}_{ij}$ or J_{ij} (see Fig. 8, Panel A in Fig. G) neither at short range nor at long range but, by capturing a collective mode, measures both to a more similar extent. By including the conservation weight ϕ_i the result is slightly improved w.r.t. the principal components of the uncorrected covariance, see Panels G, H, I in Fig. F; on the other hand, conservation only gives a particularly poor estimation regardless of the range (Panel B Fig. G). We have also tested the recent proposal by Wang et al. [46] of identifying groups of co-evolving amino acids by the top component of the inverse covariance matrix (where diagonal elements are removed before diagonalization); this method is called ICOD (Inverse Covariance Off-Diagonal). This top eigenvalue (see Panel D Fig. F) corresponds to a non-local mode mainly generated by links close to the active and allosteric site (see Panel E Fig. F) and correlates to long-range epistasis to a larger extent than previous methods, see Panel F Fig. F.

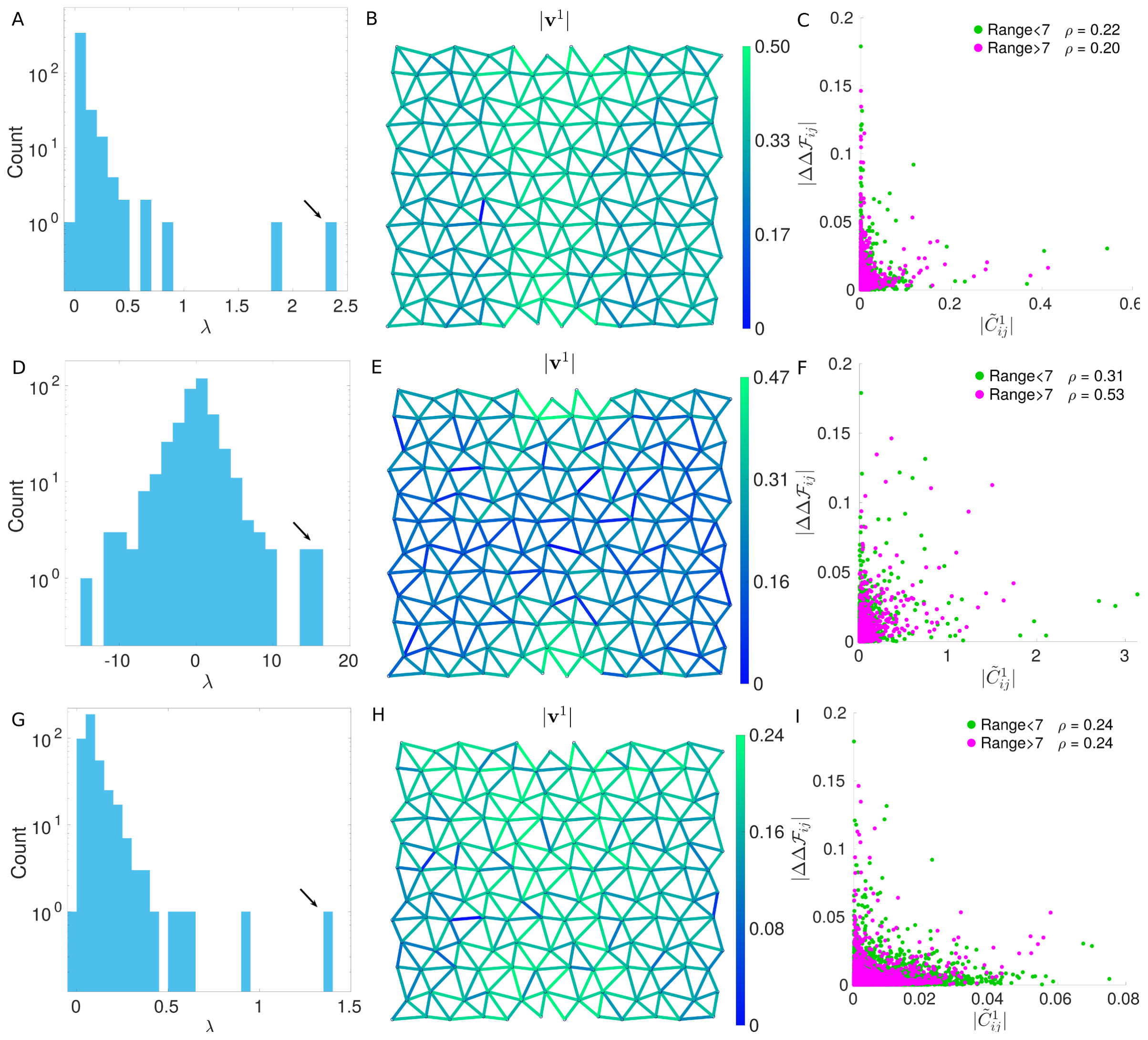

Figure F: **Measure of epistasis by SCA with conservation weight (top row A, B, C), by ICOD (central row D, E, F) and by SCA without conservation weight (bottom row G, H, I)**. A, D, G: Spectrum of eigenvalues $\boldsymbol{\lambda}$ of the conservation-weighted covariance (A), of the inverse off-diagonal covariance (D) and of the covariance itself (G), where the highest value λ^1 (corresponding to the first principal component $\boldsymbol{v}^1$) is highlighted by an arrow. B, E, H: Absolute values of the first principal component $\boldsymbol{v}^1$ visualized on the network (the first principal component of the conservation-weighted covariance in B, of the inverse off-diagonal covariance in E and of the covariance itself in H). C, F, I: Scatter plot of $\tilde{C}^1_{ij}$ vs epistasis with a different color code for long and short distance pairs, where ρ is the Pearson correlation coefficient. $\tilde{C}^1_{ij}$ is constructed from the first top eigenvalue λ^1 and its corresponding principal component $\boldsymbol{v}^1$ of the conservation-weighted covariance in C, of the inverse off-diagonal covariance in F and of the covariance itself in I.

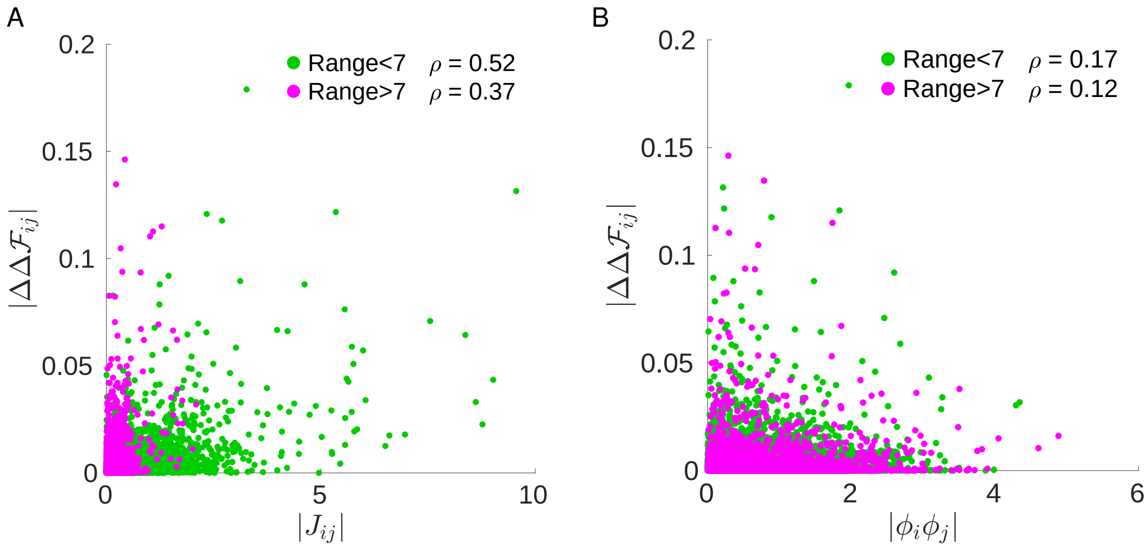

Figure G: **Epistasis measure by couplings and conservation**. A: Scatter plot of J_{ij} vs epistasis, where ρ, the Pearson correlation coefficient, indicates a better prediction at short range. B: Scatter plot of the first mode of the conservation-only matrix $\phi_i\phi_j$ vs epistasis.
